# Characterizing Genetic Risk at Known Prostate Cancer Susceptibility Loci in African Americans

**DOI:** 10.1371/journal.pgen.1001387

**Published:** 2011-05-26

**Authors:** Christopher A. Haiman, Gary K. Chen, William J. Blot, Sara S. Strom, Sonja I. Berndt, Rick A. Kittles, Benjamin A. Rybicki, William B. Isaacs, Sue A. Ingles, Janet L. Stanford, W. Ryan Diver, John S. Witte, Stephen J. Chanock, Suzanne Kolb, Lisa B. Signorello, Yuko Yamamura, Christine Neslund-Dudas, Michael J. Thun, Adam Murphy, Graham Casey, Xin Sheng, Peggy Wan, Loreall C. Pooler, Kristine R. Monroe, Kevin M. Waters, Loic Le Marchand, Laurence N. Kolonel, Daniel O. Stram, Brian E. Henderson

**Affiliations:** 1 Department of Preventive Medicine, Keck School of Medicine and Norris Comprehensive Cancer Center, University of Southern California, Los Angeles, California, United States of America; 2 International Epidemiology Institute, Rockville, Maryland, United States of America; 3 Division of Epidemiology, Department of Medicine, Vanderbilt Epidemiology Center, Vanderbilt University, Nashville, Tennessee, United States of America; 4 Vanderbilt-Ingram Cancer Center, Nashville, Tennessee, United States of America; 5 Department of Epidemiology, The University of Texas M. D. Anderson Cancer Center, Houston, Texas, United States of America; 6 Division of Cancer Epidemiology and Genetics, National Cancer Institute, National Institutes of Health, Bethesda, Maryland, United States of America; 7 Department of Medicine, University of Illinois at Chicago, Chicago, Illinois, United States of America; 8 Department of Biostatistics and Research Epidemiology, Henry Ford Hospital, Detroit, Michigan, United States of America; 9 James Buchanan Brady Urological Institute, Johns Hopkins Hospital and Medical Institutions, Baltimore, Maryland, United States of America; 10 Division of Public Health Sciences, Fred Hutchinson Cancer Research Center, Seattle, Washington, United States of America; 11 Epidemiology Research Program, American Cancer Society, Atlanta, Georgia, United States of America; 12 Institute for Human Genetics, Departments of Epidemiology and Biostatistics and Urology, University of California San Francisco, San Francisco, California, United States of America; 13 Department of Urology, Northwestern University, Chicago, Illinois, United States of America; 14 Epidemiology Program, Cancer Research Center, University of Hawaii, Honolulu, Hawaii, United States of America; University of Geneva Medical School, Switzerland

## Abstract

GWAS of prostate cancer have been remarkably successful in revealing common genetic variants and novel biological pathways that are linked with its etiology. A more complete understanding of inherited susceptibility to prostate cancer in the general population will come from continuing such discovery efforts and from testing known risk alleles in diverse racial and ethnic groups. In this large study of prostate cancer in African American men (3,425 prostate cancer cases and 3,290 controls), we tested 49 risk variants located in 28 genomic regions identified through GWAS in men of European and Asian descent, and we replicated associations (at p≤0.05) with roughly half of these markers. Through fine-mapping, we identified nearby markers in many regions that better define associations in African Americans. At 8q24, we found 9 variants (p≤6×10^−4^) that best capture risk of prostate cancer in African Americans, many of which are more common in men of African than European descent. The markers found to be associated with risk at each locus improved risk modeling in African Americans (per allele OR = 1.17) over the alleles reported in the original GWAS (OR = 1.08). In summary, in this detailed analysis of the prostate cancer risk loci reported from GWAS, we have validated and improved upon markers of risk in some regions that better define the association with prostate cancer in African Americans. Our findings with variants at 8q24 also reinforce the importance of this region as a major risk locus for prostate cancer in men of African ancestry.

## Author Summary

Prostate cancer is one of the most common cancers in men and is especially frequent in men of African origin, as incidence rates in African Americans in the United States are >1.5–fold greater than rates in European Americans. In order to gain a more complete understanding of the genetic basis of inherited susceptibility to prostate cancer in men of African origin, we examined the associations at risk loci identified in men of European and Asian descent in a large African American sample of 3,425 cases of prostate cancer and 3,290 male controls. In testing 49 known risk variants, we were able to demonstrate that at least half of these variants also contribute to risk in African American men. We were able to find additional risk variants in many of the previously reported regions that better captured the pattern of risk in African American men. In addition, we verified and improved upon the evidence we previously reported that there are multiple risk variants in a region of 8q24 that are important in men of African origin.

## Introduction

Genome-wide association studies (GWAS) have revealed more than 30 variants that contribute susceptibility to prostate cancer, with most of the discoveries having been made in populations of European ancestry [1]–[14]. However, as so far observed for most common diseases, variants identified through GWAS are of low risk both individually and in aggregate, and therefore provide only limited information about disease prediction [15], [16]. Most risk variants for prostate cancer are located outside of annotated genes, with some positioned in gene poor regions and some regions harboring more than one independent signal [1], [10], [14], [17], [18]. Thus, for the vast majority of risk loci, the identity, frequency and risk associated with the underlying biologically relevant allele(s) are unknown. The risk variants revealed through GWAS have also been found to vary in frequency across racial/ethnic populations [19]. Even in the absence of functional data, the associated risk variants may highlight a genetic basis for differences in disease risk between populations, such as at 8q24 where genetic variation is suggested to contribute to population differences in risk of prostate cancer [10]. Testing of the risk variants and fine-mapping in diverse populations will help to identify and localize the subset of markers that best define risk of the functional allele(s) within known risk loci, as well as to determine their contribution to racial and ethnic differences in prostate cancer risk.

In the present study, we tested common genetic variation at the prostate cancer risk loci identified in men of European and Asian descent in a large sample comprised of 3,425 African American prostate cancer cases and 3,290 controls, to identify markers of risk that are relevant to this population. More specifically, we conducted GWAS and imputation-based fine-mapping of each risk locus to both improve the current set of risk markers in African Americans as well as to identify new risk variants for prostate cancer. We then applied this information to model the genetic risk of prostate cancer in African American men.

## Results

The African American prostate cancer cases (n = 3,621) and controls (n = 3,502) in this study are part of a collaborative genome-wide scan of prostate cancer that includes 11 individual studies (Table S1, Methods). Samples were genotyped using the Illumina Infinium 1M-Duo bead array, and following quality control exclusions (see Methods), the analysis of variants at the known risk loci was performed on 3,425 cases and 3,290 controls. The ages of cases and controls ranged from 23 to 95, with cases and controls having similar ages (mean 65 and 64 years, respectively).

**Figure 1:**
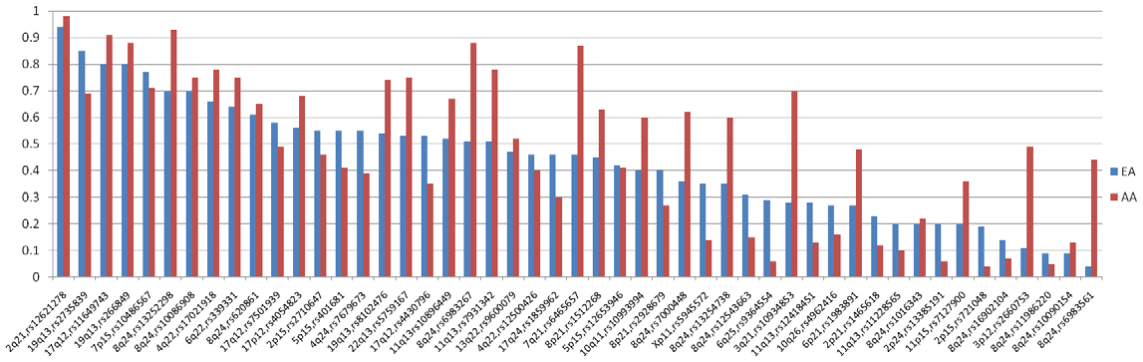
Risk Allele Frequencies in Europeans and African Americans. The distribution of risk allele frequencies (RAF) for the 49 index SNPs (from Table 1 and Table 2) in Europeans (EA) and African Americans (AA). The variants are sorted based on the RAF in EAs.

We tested 49 known prostate cancer risk variants located in 28 risk regions (Table S2, Table 1, and Table 2); 43 SNPs were directly genotyped (with call rates >95%), while 6 were imputed with high accuracy (see Methods) [1], [3], [4], [6]–[14], [17], [18], [20]–[23]. The minor allele frequencies (MAF) of all 49 variants were common (≥0.05) in African Americans, except for rs721048 at 2p15 (MAF, 0.04) and rs12621278 at 2q21 (MAF, 0.02; Table 1, Figure 1). On average, across all variants tested, the risk allele frequencies (RAFs, i.e. alleles associated with an increased risk of prostate cancer in previous GWAS) were 0.05 greater in African Americans than in Europeans. However, when removing the 12 risk variants at 8q24 (Table 2) the average difference in RAF over the remaining risk loci was only 0.03.

**Table 1:** Associations with common variants at known prostate cancer risk regions in African Americans (3,425 cases, 3,290 controls).

Index SNP from GWAS		Best Marker in African Americans		
Chr., Marker Position, Alleles^a^	RAF (EA/AA)^b^, OR (95% CI)^c^P-value^d^	Marker, Position, Alleles^a^	RAF (AA)^b^, OR (95% CI)^c^P-value	r^2^ with index in GWAS population/YRI^e^
2p24,rs13385191 20,751,746,G/A	0.61^f^/0.06, 0.99(0.84–1.16) 0.90	rs340623^g^ 20,795,759,C/T	0.17, 1.15(1.05–1.27) 3.8×10^−3^	0.44^k^/0^h^
2p21,rs1465618 43,407,453,T/C	0.23/0.12, 1.07(0.96–1.20) 0.22	-----j		
2p15, rs721048 62,985,235,A/G	0.19/0.04, 1.24(1.03–1.50) 0.025	-----		
2p15,rs2710647 63,067,474,C/T	0.55/0.46, 1.16(1.08–1.25) 2.8×10^−5^	rs6545977 63,154,668,G/A	0.48, 1.18(1.10–1.27) 2.3×10^−6^	0.42/0.44
2q21,rs12621278 173,019,799,A/G	0.94/0.98, 1.44(1.05–1.99) 0.026	rs12620581^g^ 173,037,960,A/G	0.75, 1.13(1.04–1.23) 3.8×10^−3^	0.29/0^h^
3p12,rs2660753 87,193,364,T/C	0.11/0.49, 0.97(0.90–1.05) 0.50	-----		
3q21,rs10934853 129,521,063,A/C	0.28/0.70, 1.03(0.95–1.13) 0.43	rs7641133 129,319,009,T/C	0.29, 1.16(1.08–1.25) 1.0×10^−4^	0.91/0.11
4q22,rs12500426 95,733,632,A/C	0.46/0.40, 1.00(0.93–1.07) 0.99	-----		
4q22,rs17021918 95,781,900,C/T	0.66/0.78, 1.08(0.99–1.18) 0.066	-----		
4q24,rs7679673^g^ 106,280,983,C/A	0.55/0.39, 1.08(1.00–1.16) 0.050	-----		
5p15,rs401681 1,375,087,C/T	0.55/0.41, 0.94(0.87–1.00) 0.068	-----		
5p15,rs12653946 1,948,829,T/C	0.43^f^/0.41, 1.05(0.98–1.13) 0.15	-----		
6p21,rs1983891 41,644,405,T/C	0.38^f^/0.48, 1.09(1.01–1.17) 0.024	-----		
6q22,rs339331 117,316,745,T/C	0.63^f^/0.75, 1.22(1.12–1.32) 3.1×10^−6^	rs12202378^g^ 117,348,714,T/C	0.70, 1.25(1.15–1.35) 8.8×10^−8^	1.0^k^/0.79
6q25,rs9364554 160,753,654,T/C	0.29/0.06, 1.30(1.11–1.52) 8.2×10^−4^	rs2076828 160,792,776,C/G	0.56, 1.14(1.06–1.22) 3.5×10^−4^	0.29/0^h^
7p15,rs10486567 27,943,088,G/A	0.77/0.71, 1.15(1.07–1.25) 2.9×10^−4^	rs7808935^g^ 27,943,888,T/C	0.70, 1.16(1.07–1.25) 2.6×10^−4^	0.93/1.0
7q21,rs6465657 97,654,263,C/T	0.46/0.87, 1.00(0.87–1.14) 0.95	-----		
8p21,rs2928679 23,494,920,A/G	0.42/0.27, 1.02(0.94–1.10) 0.60	-----		
8p21,rs1512268 23,582,408,T/C	0.45/0.63, 1.12(1.04–1.20) 3.2×10^−3^	rs11782388^g^ 23,581,303,C/T	0.70, 1.18(1.09–1.28) 9.8×10^−5^	0.95/0.63
10q11,rs10993994 51,219,502,T/C	0.40/0.60, 1.09(1.02–1.17) 0.017	rs4630243^g^ 51,210,873,T/C	0.76, 1.14(1.05–1.25) 2.3×10^−3^	0.74/0.27
10q26, rs4962416 126,686,862,C/T	0.27/0.16, 1.05(0.96–1.16) 0.28	-----		
11p15, rs7127900 2,190,150,A/G	0.20/0.36, 1.09(1.01–1.17) 0.027	-----		
11q13,rs12418451^g^ 68,691,995,A/G	0.28/0.13, 1.13(1.01–1.27) 0.030	-----		
11q13,rs11228565 68,735,156,A/G	0.20/0.10, 1.08(0.96–1.21) 0.18	rs11228580^g^ 68,758,918,C/T	0.16, 1.31(1.20–1.44) 9.7×10^−9^	0.53/0.05
11q13, rs7931342 68,751,073,G/T	0.51/0.78, 1.13(1.03–1.24) 8.9×10^−3^	-----		
11q13,rs10896449 68,751,243,G/A	0.52/0.67, 1.15(1.06–1.24) 3.7×10^−4^	-----		
13q22,rs9600079 72,626,140,T/G	0.35^f^/0.52, 0.98(0.91–1.05) 0.53	-----		
17p12, rs4054823 13,565,749,T/C	0.56/0.68, 0.99(0.92–1.06) 0.74	-----		
17q12,rs11649743 33,149,092,G/A	0.80/0.91, 1.15(1.01–1.31) 0.041	-----		
17q12, rs4430796 33,172,153,A/G	0.53/0.35, 1.02(0.95–1.10) 0.52	-----		
17q12,rs7501939 33,175,269,C/T	0.58/0.49, 1.03(0.96–1.10) 0.44	-----		
17q24, rs1859962 66,620,348,G/T	0.46/0.30, 0.99(0.92–1.07) 0.84	-----		
19q13, rs8102476 43,427,453,C/T	0.54/0.74, 1.12(1.03–1.21) 8.5×10^−3^	-----		
19q13, rs266849 56,040,902,A/G	0.80/0.88, 1.01(0.91–1.13) 0.85	rs3760722^i^ 56,049,628,C/T	0.72, 1.14(1.05–1.24) 1.5×10^−3^	0.22/0.02
19q13, rs2735839 56,056,435,G/A	0.85/0.69, 0.94(0.87–1.02) 0.12	-----		
22q13, rs5759167 41,830,156,G/T	0.53/0.75, 1.10(1.01–1.20) 0.024	-----		
Xp11, rs5945572 51,246,423,A/G	0.35/0.14, 1.21(1.09–1.35) 5.2×10^−4^	rs4907796 51,277,989,T/C	0.13, 1.25(1.12–1.39) 7.1×10^−5^	0.87/0.72

a Risk allele/reference allele.

b RAF, risk allele frequency in populations of European ancestry (EA) or HapMap CEU population, and in African Americans (AA) in this study. This is the allele associated with increased risk in previous GWAS.

c Adjusted for age, study, the 1^st^ 10 eigenvalues and local ancestry at each risk locus.

d Test of trend (1-d.f.).

e Pairwise correlation between the index signal and the best marker in African Americans in CEU or JPT (where indicated) in 1000 Genomes Project (March 2010 release).

f Index signal reported in Japanese. RAFs and r^2^ based on Japanese data [11] or JPT in 1000 Genomes.

g Imputed (Rsq≥0.87).

h Best marker or index marker in AA is extremely rare or monomorphic in YRI.

i r^2^ of rs3760722 and rs2735839 in YRI is 0.24.

j No SNP selected in stepwise procedure.

k Estimated in HapMap JPT/CHB.

**Table 2:** Associations with risk variants at 8q24 in African Americans.

African Americans (3,425 cases, 3,290 controls)							
Region^a^, Position	Marker, Alleles^b^	RAF^c^ (EA/AA)	OR (95% CI)^d^	P-value^e^	OR (95% CI) Adjusted^f^	P-value	R^2^ g
1, 127,993,841	rs12543663, C/A	0.31/0.15	0.89(0.80–0.99)	0.028	0.91(0.82–1.02)	0.10	0.07
1, 128,081,119	rs10086908, T/C	0.70/0.75	1.13(1.04–1.22)	4.5×10^−3^	1.13(1.04–1.23)	4.2×10^−3^	0.06
2, 128,162,479	rs1016343, T/C	0.20/0.22	1.03(0.95–1.12)	0.51	1.02(0.94–1.11)	0.68	0.03
2, 128,164,338	rs13252298, A/G	0.70/0.93	1.09(0.93–1.27)	0.28	1.04(0.89–1.22)	0.60	0.12
2, 128,173,525	rs13254738^h^, C/A	0.35/0.60	1.25(1.16–1.36)	2.1×10^−8^	1.17(1.07–1.28)	7.3×10^−4^	0.31
2, 128,176,062	rs6983561^h^, C/A	0.04/0.44	1.29(1.19–1.39)	5.6×10^−10^	1.20(1.09–1.31)	1.0×10^−4^	0.33
3, 128,404,855	rs620861, G/A	0.61/0.65	1.06(0.99–1.14)	0.11	1.07(0.99–1.15)	0.088	0.06
3, 128,410,090	rs16902104, T/C	0.14/0.07	1.01(0.88–1.16)	0.88	0.97(0.84–1.12)	0.72	0.05
4, 128,482,487	rs6983267, G/T	0.51/0.88	1.24(1.09–1.42)	1.5×10^−3^	1.20(1.04–1.38)	0.011	0.21
4, 128,510,352	rs7000448^h^, T/C	0.36/0.62	1.11(1.02–1.20)	0.012	1.08(0.99–1.18)	0.070	0.16
5, 128,600,871	rs11986220^h^, A/T	0.09/0.05	1.39(1.20–1.61)	1.5×10^−5^	1.28(1.06–1.56)	0.011	0.42
5, 128,601,319	rs10090154^h^, T/C	0.09/0.13	1.22(1.10–1.35)	2.0×10^−4^	1.08(0.95–1.24)	0.24	0.42

Stepwise Analysis^i^							
Region^a^, Position	Marker, Alleles^b^	RAF^c^ (EA/AA)	OR (95% CI)^d^	P-value^e^	OR (95% CI) Adjusted^f^	P-value	R^2^ g
1,127,994,810	rs7839365^h^, T/A	0.60/0.61	1.18(1.09–1.27)	1.7×10^−5^	1.16(1.08–1.26)	1.1×10^−4^	0.01
1,128,059,437	rs753228^h^, C/T	0.96/0.95	1.41(1.18–1.68)	1.5×10^−4^	1.43(1.20–1.72)	9.9×10^−5^	0.02
2,128,162,723	rs4871008^h^, C/T	0.57/0.67	1.19(1.10–1.28)	7.4×10^−6^	1.15(1.06–1.24)	7.9×10^−4^	0.10
2,128,173,119	rs1456315, T/C	0.28/0.53	1.23(1.15–1.33)	8.0×10^−9^	1.27(1.18–1.37)	4.2×10^−10^	0.06
2,128,200,973	rs10098156^h^, G/C	0.90/0.88	1.26(1.11–1.44)	4.9×10^−4^	1.30(1.13–1.49)	2.2×10^−4^	0.09
2,128,219,343	rs6987409^h^, T/C	0.0/0.15	1.42(1.28–1.57)	1.8×10^−11^	1.33(1.20–1.48)	1.1×10^−7^	0.08
4,128,528,307	rs13282506^h^, G/A	0.73/0.88	1.25(1.09–1.43)	1.3×10^−3^	1.28(1.12–1.47)	3.8×10^−4^	0.006
5,128,589,355	rs7812429^h^, A/G	0.06/0.08	1.31(1.15–1.48)	3.4×10^−5^	1.30(1.15–1.48)	5.0×10^−5^	0.002
5,128,640,941	rs4313118, T/C	0.77/0.79	1.16(1.07–1.27)	6.2×10^−4^	1.17(1.07–1.28)	4.1×10^−4^	0.001

a Risk regions as defined in [1], [2], [7], [10], [13].

b Risk /reference alleles.

c RAF, risk allele frequency in populations of European ancestry [1], [6 or HapMap CEU] and in African Americans (AA).

d Adjusted for age, study, the 1^st^ 10 eigenvalues and local ancestry for region 127.8–129.0 Mb (NCBI build 36).

e Test of trend (1-d.f.).

f From the multivariate model. OR adjusted for age, study, the 1^st^ 10 eigenvalues, local ancestry and all other 8q24 risk variants.

g The proportion of the variance explained by the other SNPs.

h Imputed (Rsq≥0.76). rs445114 was not genotyped and could not be imputed [6].

i SNPs kept in stepwise procedure if p<0.001.

We examined the association of local ancestry with prostate cancer risk at each of the 28 risk regions (Table S3). In addition to 8q24, which we had previously found to be strongly associated with African ancestry [5] (OR per European chromosome = 0.81, p = 4.7×10^−5^), we observed significant associations at 22q13 (OR = 0.88, p = 0.01), 7p15 (OR = 1.16, p = 1.6×10^−3^) and 10q26 (OR = 1.14, p = 6.2×10^−3^). To address the potential for confounding by genetic ancestry, we adjusted for both global and local ancestry in all analyses (see Methods).

In previous GWAS, the index signals outside of 8q24 had very modest odds ratios (1.05–1.30 per copy of the risk allele) and our sample size provided ≥80% power to detect the reported effects for 24 of the 37 variants (at p<0.05; Table S2). We observed positive associations with 28 of the 37 variants (odds ratios (OR) >1) in African Americans and 18 reached nominal statistical significance (p≤0.05; Table 1). Results were similar without adjustment for local ancestry in each region (Table S4). Of the 19 variants that were not replicated at p<0.05, power was <80% for 9 of the variants.

While power was limited to detect associations at some loci, the lack of replication at loci where power was acceptable (>80%) suggests that the particular risk variant revealed in GWAS in European and Asian populations may not be adequately correlated with the biologically relevant allele in African Americans. In an attempt to identify a better genetic marker of the biologically relevant allele in African Americans we conducted fine-mapping across all risk regions using genotyped SNPs on the 1 M array and imputed SNPs to Phase 2 HapMap (Table S5, see Methods). If a marker associated with risk in African Americans represents the same signal as that reported in the initial GWAS, then it should be correlated to some degree with the index signal in the initial GWAS population. Using HapMap data (CEU or JPT+CHB depending upon the initial GWAS population) we catalogued and tested all SNPs that were correlated (r^2^≥0.2) with the index signal (within 250 kb), applying a significance criteria α_a_, of 0.004 given the large number of correlated tests. This level of significance was based on the number tag SNPs in the HapMap YRI population that capture (r^2^≥0.8) all SNPs that were correlated with the index signal in the HapMap CEU (r^2^≥0.2; see Methods). We also looked for novel independent associations, focusing on the genotyped and imputed SNPs that were uncorrelated with the index signal in the initial GWAS populations. Here, we applied a Bonferroni correction for defining novel associations as significant in each region, with α_b_ estimated as 0.05/the total number of tags needed to capture (r^2^≥0.8) all common risk alleles across all risk region in the YRI population (α_b_ = 5.6×10^−6^). This is similar to the genome-wide-type correction of 5×10^−8^, which accounts for the number of tags needed to capture all common alleles in the genome. For each region, stepwise regression was used with SNPs kept in the final model based on α_a_ or α_b_ (results for each model are provided in Table S6).

**Figure 2:**
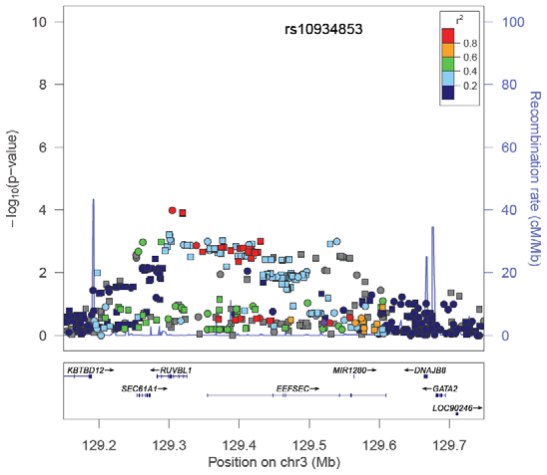
−Log P Plot for Common Alleles at the Chromosome 3q21 Prostate Cancer Risk Locus. The index signal (rs10934853) is designated by a purple diamond. The r^2^ shown is that in Europeans from HapMap (CEU) in relation to rs10934853. −Log P-values are those observed in African Americans from logistic regression models adjusted for age, study, global ancestry (the 1^st^ 10 eigenvectors) and local ancestry. Circles are genotyped SNPs and squares are imputed SNPs. Grey circles are SNPs not in HapMap (r^2^ can not be estimated). The plot was generate using LocusZoom [45].

Among the SNPs correlated with the index signal in the GWAS population, a more significantly associated marker was identified at 12 regions. For 5 of these regions, the new marker showed only a slightly more significant association than the index signal (<1 order of magnitude change in the p-value; Table 1). However, for 7 regions (2p24, 2p15, 3q21, 6q22, 8q21, 11q13, and 19q13), the new marker appeared to capture risk more strongly than the index signal in African Americans. The risk region at 3q21 is provided in Figure 2 as an example. Here the index signal was not significantly associated with risk in African Americans (rs10934853, OR = 1.03, 95% CI, 0.95–1.03, p = 0.43), with the most significantly associated marker in African Americans located ∼200 kb centromeric from the index signal (rs7641133, OR = 1.16, 95% CI 1.08–1.25, p = 1.0×10^−4^). These two markers are strongly correlated in Europeans (HapMap CEU, r^2^ = 0.91) but not in Africans (HapMap YRI, r^2^ = 0.11; Table 1), which suggests that in African Americans rs7641133 is a better proxy of the biologically important allele and may better localize the true association. For some of these regions, the size of the LD blocks differ in populations of African ancestry compared with the GWAS population and thus, may assist in localizing the functional allele (Figure S1). Using a strict α_b_ of 5.6×10^−6^ for discovery of novel risk variants we observed no evidence of a second independent signal at any risk region. For variants identified as significantly associated with risk (Table 1), odds ratios for homozygous carriers were generally greater than for heterozygous carriers, which provides support for their associations (Table S7).

We examined 12 risk variants at 8q24 that had been reported previously to be associated with prostate cancer risk [1], [7], [10], [13], [14], [20] with 7 being statistically significant and positively associated with risk (p<0.05). The risk SNP BD11934905 [10] is not on the Illumina 1 M array and was not genotyped in this study. In contrast with what has been reported in Europeans, the risk allele for rs12543663 was observed to be significantly inversely associated with risk in African Americans (OR = 0.89, p = 0.028; Table 2). The RAFs for 8 of the 12 alleles are more common in African Americans than Europeans, with the average RAF being 0.46 in African Americans and 0.32 in Europeans. The largest difference in RAFs between populations are noted with variants rs13252298, rs13254738, rs6983561, rs6983267 and rs7000448, which have RAFs that are >0.20 greater in African Americans than in Europeans. When all 12 variants were included in a multivariate model, only 5 remained nominally associated with risk (Table 2). In African Americans, many of these index signals were weakly correlated (Figure S2) and demonstrated stronger multi-allelic correlations (Table 2), which suggests that some variants may define similar haplotypes marking the same biologically relevant variants in this population. No significant association was observed with rs7008482 (OR = 0.96, p = 0.52, computed using data included in the initial report [24]) or markers of risk at 8q24 for cancers of the breast, bladder, ovary, or leukemia (rs13281615: OR = 1.03, p = 0.48; rs9642880: OR = 1.07, p = 0.13; rs10088218: OR = 0.91, p = 0.06; rs2456449: OR = 1.06, p = 0.24) [25]–[28].

**Figure 3:**
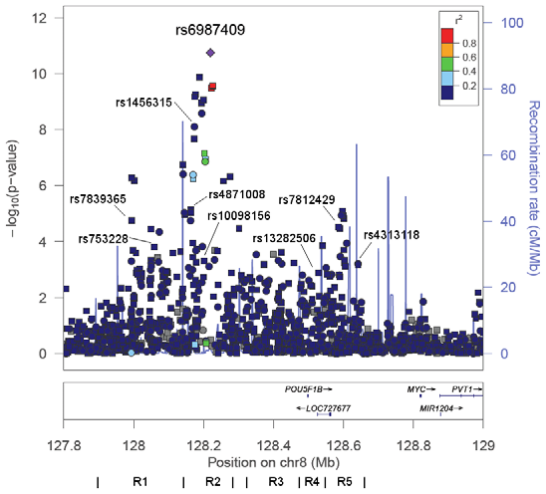
−Log P Plot for Common Alleles at 8q24 in African Americans. −Log P-values for alleles in the region 127.8–129.0 Mb in African Americans from logistic regression models adjusted for age, study, global ancestry (the 1^st^ 10 eigenvectors) and local ancestry. Pairwise correlations in the HapMap YRI population are shown in relation to rs6987404, which was the most significant marker in the region (p = 1.8×10^−11^). Circles are genotyped SNPs and squares are imputed SNPs. Grey circles are SNPs not in HapMap (r^2^ can not be estimated). The lines below demarcate the five risk regions (R) as defined in [1], [2], [7], [10], [13]. The plot was generate using LocusZoom [45]. The nine SNPs highlighted are from the stepwise analysis presented in Table 2.

To identify markers at 8q24 that best capture risk in African Americans we performed a stepwise analysis of 1,549 genotyped and imputed SNPs spanning the established risk locus (127.8–129.0 Mb). This region contained 132 SNPs with nominal p-values<0.001 (Figure 3), and 9 common alleles with per allele ORs of 1.16–1.42 (Table 2) defined the most parsimonious model. Similarly to the previously reported risk variants at 8q24 four of these markers are substantially more common in African Americans than Europeans (average RAF difference = 0.07). Eight of these markers show some degree of correlation with the known risk variants and thus are likely to be tagging the same functional allele, albeit for 4 SNPs the correlations are quite weak in the CEU and YRI populations (r^2^<0.2; Table S8) suggesting that they may be marking independent risk variants. For example, SNP rs6987409 (RAF = 0.15), which is monomorphic in Europeans, remains significantly associated with risk conditional on the 12 known risk alleles at 8q24 (OR = 1.31, 95% CI, 1.16–1.47, p = 7.1×10^−6^), which suggests that this SNP may be marking a novel variant that is relevant in African Americans; rs6987409 was the most significant marker in the region (Figure 3).

We next estimated the cumulative effect of all prostate cancer risk alleles, and compared a summary risk score comprised of unweighted counts of all GWAS reported risk alleles to a risk score that included variants we identified as being associated with risk in African Americans (Table 3). Using index signals from GWAS (see Methods), the risk per allele was 1.08 (95% CI, 1.06–1.09; p = 6.0×10^−26^) and individuals in the top quartile of the risk allele distribution were at 2-fold greater risk of prostate cancer compared to those in the lowest quartile (Table 3). As expected, the risk score was improved when utilizing the markers that we identified at the known risk loci as being more relevant to African Americans (OR = 1.17 95% CI, 1.15–1.19; p = 5.1×10^−74^), with risk for those in the top quartile being 3.5-times those in the lowest quartile. When stratifying by first-degree family history of prostate cancer, risk was 4.7-fold greater for those with a family history and in the top quartile of the risk score distribution (3.5% of the population) compared to those without a family history and in the first quartile (Table 3). The risk score was associated equally with risk for advanced (n = 1,087) and non-advanced (n = 1,968) prostate cancer (case-only test: OR = 1.02, 95% CI, 1.00–1.05 p_het_ = 0.082).

**Table 3:** The association of the total risk score with prostate cancer risk in African Americans.

		Index Markers from GWAS (n = 40)	Risk-associated Markers in African Americans (n = 27)		
Mean number of risk alleles in controls, (range)		41(24–54)	31(20–43)		
OR per allele (95% CI)^a^		1.08(1.06–1.09)	1.17(1.15–1.19)		
P-value		6.0×10^−26^	5.1×10^−74^		
			All cases/controls(3425/3290)	First-Degree Family History Negative^c^(2505/2454)	First-Degree Family History Positive^c^(574/317)
Quartiles of Risk Alleles^b^					
Q1	n (cases/controls)	603/824	441/834	328/610	66/92
	OR(95% CI)	1.0(ref.)	1.0(ref.)	1.0(ref.)	1.19(0.83–1.72)
	P-value	-	-	-	0.34
Q2	n (cases/controls)	775/915	717/853	530/615	122/69
	OR(95% CI)	1.16(1.00–1.34)	1.60(1.37–1.87)	1.50(1.25–2.18)	3.00(2.14–4.22)
	P-value	0.05	4.6×10^−9^	1.8×10^−5^	2.1×10^−10^
Q3	n (cases/controls)	841/732	804/795	601/598	128/69
	OR(95% CI)	1.55(1.33–1.80)	1.89(1.62–2.21)	1.81(1.51–2.18)	2.94(2.10–4.12)
	P-value	1.0×10^−8^	1.1×10^−15^	2.8×10^−10^	3.8×10^−10^
Q4	n (cases/controls)	1206/823	1463/808	1046/591	258/87
	OR(95% CI)	2.02(1.75–2.33)	3.51(3.02–4.07)	3.33(2.79–3.97)	4.66(3.48–6.23)
	P-value	9.4×10^−22^	6.9×10^−61^	1.6×10^−40^	3.4×10^−25^

a Odds ratios (and 95% confidence intervals) adjusted for age, study, and the 1^st^ 10 eigenvalues.

b Quartiles based on distribution in controls (cutpoints for 40 SNPs: 37.5, 40.0 and 42.7; 27 SNPs: 28.7, 30.9 and 32.8).

c Information about family history of prostate cancer is available on 90% of cases and 84% of controls.

Using this risk score, we estimate (see Methods) that in the aggregate, all risk alleles tested explain approximately 11% of risk in first-degree relatives of cases.

## Discussion

In this large study of prostate cancer risk in African American men we tested 49 variants that had been reported primarily in populations of European and Asian ancestry, and we were able to replicate associations (at p≤0.05) with roughly half of these markers. We had adequate power (>80%) to detect relative risks of the magnitude reported previously for the majority of risk variants (although we realize that power was overestimated as the effect estimates from the initial report may be inflated due to the winner's curse phenomenon [29].) Through fine-mapping, we identified markers in many regions that were more strongly associated with risk in African Americans than the index variant, and thus, are likely to be better proxies of the biologically relevant allele in this population. Our ability to detect associations in African Americans with either the index signal or correlated variants suggests that most loci contain a biologically relevant allele that is not unique to the initial GWAS population. These findings improve upon previous studies to replicate associations in African Americans [30], efforts which included some of these same studies, but in substantially smaller sample sizes for most variants examined [19], [31].

Within 12 regions, fine-mapping in African Americans revealed a more significantly associated marker (with evidence over the index signal being clearly greater at 7 loci). For some of the regions, the signal in African Americans was located in a smaller region of LD than that observed in the GWAS population which should aid in localizing the functional variant(s). Confirmation of these associations in the initial GWAS populations will be required before they can be declared as proxies of the underlying functional alleles; however in many cases, given their modest to strong correlation, based on HapMap data, with the index signal in the GWAS population, most markers are expected to be strongly associated with risk. At each locus, fine-mapping was based on the Illumina 1 M-Duo content supplemented with SNPs imputed from Phase 2 HapMap (CEU/YRI), which is expected to provide good coverage of the vast majority of common alleles in the admixed African American population. Of the ∼1.5 million common SNPs (MAF≥0.05) in the HapMap YRI population that we did not genotype, we were able to impute ∼1.4 million with Rsq≥0.8. Our inability to detect associations at 10 regions (p>0.05 for an index signal and p>0.004 for a proxy) could be due to low power, the functional allele being rare or non-existent in African Americans and/or inadequate tagging in these specific regions.

Because of limited LD, fine-mapping in African Americans is thought to be an effective approach for localizing functional risk alleles for common phenotypes as populations of African ancestry are expected to have, on average, fewer alleles that are correlated with a functional variant. Fine-mapping in multiple racial/ethnic populations should prove to be even more powerful for isolating these variants as only a subset SNPs that are correlated with the functional allele in different populations will be similar. Thus, conducting association testing across multiple populations should narrow the subset of potentially functional alleles in a region. A complete resource of genome-wide variation data from multiple populations provided by the 1000 Genomes Project will assist in further interrogating these risk loci and together with large-scale association testing in diverse samples, will guide researchers in defining the subset of alleles that are correlated with risk across populations and hence are the most logical candidates for functional characterization.

A number of prostate cancer risk regions have been found to harbor more than one risk variant (e.g. 8q24, 17q12 and 11q13) [1], [10], [17], [18]. Aside from 8q24, the search for independent markers at known risk loci has been limited to populations of European ancestry. Using a relatively strict threshold for declaring significance (average α<5.6×10^−6^), we observed no evidence of association that is independent of the index signal. While suggestive associations were observed at many loci, testing of these variants in additional African American samples will be needed to confirm these associations, followed by testing in other populations to assess whether the associations may be limited to African Americans.

The risk region at 8q24 is the strongest susceptibility locus for prostate cancer that has been identified to date, with a number of different risk variants having been reported in different populations [1], [6], [7], [10], [13], [14]. We identified nine SNPs at 8q24 that best captured the genetic risk in African Americans, including SNP rs6987409 [1] which is not observed in Europeans (or is present at an extremely low frequency). Like the reported index signals at 8q24 (Table 2), many of these markers are more common in African Americans than in Europeans (average RAF difference = 0.07). This is in contrast to the index signals in regions outside of 8q24 where the RAF average difference was only 0.03. If the frequency of these 8q24 variants is a good correlate of the frequency of the underlying biologically relevant alleles then some of the variants in this region may to contribute to the excess risk of prostate cancer in African Americans, as suggested previously [10]. A precise estimate of its contribution will only come once the functional alleles have been found and we understand their associations in the context of other genetic and environmental factors (or host factors such as age).

The cumulative effects of GWAS-identified variants for common cancers are not yet clinically informative for risk prediction [15], [16]. Until the functional alleles are identified and their effects are accurately estimated, modeling of the genetic risk will rely on markers that best capture risk at an established susceptibility locus for a given population. Many of the markers we identified at these risk loci in African Americans appear to provide substantial improvement over the GWAS-identified variants in defining those who are at greater risk of prostate cancer in this population. However, as estimated with the index signals in European populations [3], these alleles likely account for only a small fraction of the familial risk of the disease (∼10%) in African Americans. Validation of this risk model in African Americans and in other populations will be needed, as will incorporating novel risk variants identified through this GWAS in African American men.

## Methods

### Ethics Statement

The Institutional Review Board at the University of Southern California approved the study protocol.

### Study Populations

Nine studies were genotyped as part of the GWAS of prostate cancer in African American men. Below is a brief description of each study.

#### The Multiethnic Cohort (MEC)

The MEC includes 215,251 men and women aged 45–75 years at recruitment from Hawaii and California [32]. The cohort was assembled in 1993–1996 by mailing a self-administered, 26-page questionnaire to persons identified primarily through the driver's license files. Identification of incident cancer cases is by regular linkage with the Hawaii Tumor Registry and the Los Angeles County Cancer Surveillance Program; both NCI-funded Surveillance, Epidemiology, and End Results registries. From the cancer registries, information is obtained about stage and grade. Collection of biospecimens from incident prostate cases began in California in 1995 and in Hawaii in 1997 and a biorepository was established between 2001 and 2006 from 67,000 MEC participants. The participation rates for providing a blood sample have been greater than 60%. Through January 1, 2008 the African American case-control study in the MEC included 1,094 cases and 1,096 controls.

#### The Southern Community Cohort Study (SCCS)

The SCCS is a prospective cohort of African and non-African Americans which during 2002–2009 enrolled approximately 86,000 residents aged 40–79 years across 12 southern states [33]. Recruitment occurred mainly at community health centers, institutions providing basic health services primarily to the medically uninsured, so that the cohort includes many adults of lower income and educational status. Each study participant completed a detailed baseline questionnaire, and nearly 90% provided a biologic specimen (approximately 45% a blood sample and 45% buccal cells). Follow-up of the cohort is conducted by linkage to national mortality registers and to state cancer registries. Included in this study are 212 incident African American prostate cancer cases and a matched stratified random sample of 419 African American male cohort members without prostate cancer at the index date selected by incidence density sampling.

#### The Prostate, Lung, Colorectal, and Ovarian Cancer Screening Trial (PLCO)

The Prostate, Lung, Colorectal, and Ovarian Cancer Screening Trial [34], is a randomized, two-arm trial among men and women aged 55–74 years to determine if screening reduced the mortality from these cancers. Male participants randomized to the intervention arm underwent prostate specific antigen (PSA) screening at baseline and annually for 5 years and digital rectal examination at baseline and annually for 3 years. Sequential blood samples were collected from participants assigned to the screening arm; participation was 93% at the baseline blood draw (1993–2001). Buccal cell samples were collected from participants in the control arm of the trial; participation was about 85% for this component. Included in this study are 286 African American prostate cancer cases and 269 controls without a history of prostate cancer, matched on age at randomization and study year of the trial.

#### The Cancer Prevention Study II Nutrition Cohort (CPS-II)

The CPS-II Nutrition Cohort includes over 86,000 men and 97,000 women from 21 US states who completed a mailed questionnaire in 1992 (aged 40–92 years at baseline) [35]. Starting in 1997, follow-up questionnaires were sent to surviving cohort members every other year to update exposure information and to ascertain occurrence of new cases of cancer; a >90% response rate has been achieved for each follow-up questionnaire. From 1998–2001, blood samples were collected in a subgroup of 39,376 cohort members. To further supplement the DNA resources, during 2000–2001, buccal cell samples were collected by mail from an additional 70,000 cohort members. Incident cancers are verified through medical records, or through state cancer registries or death certificates when the medical record can not be obtained. Genomic DNA from 76 African American prostate cancer cases and 152 age-matched controls were included in stage 1 of the scan.

#### Prostate Cancer Case-Control Studies at MD Anderson (MDA)

Participants in this study were identified from epidemiological prostate cancer studies conducted at the University of Texas M.D. Anderson Cancer Center in the Houston Metropolitan area since 1996. Cases were accrued from six institutions in the Houston Medical Center and were not restricted with respect to Gleason score, stage or PSA. Controls were identified via random-digit-dialing or among hospital visitors and they were frequency matched to cases on age and race. Lifestyle, demographic, and family history data were collected using a standardized questionnaire. These studies contributed 543 African American cases and 474 controls to this study [36].

#### Identifying Prostate Cancer Genes (IPCG)

Cases in this study were patients 1) undergoing treatment for prostate cancer in the Department of Urology at Johns Hopkins Hospital from 1999 to 2007; 2) undergoing treatment at the Sidney Kimmel Comprehensive Cancer Center from 2003 to 2007; and 3) outside referrals as part of the Hereditary Prostate Cancer Study from 1990 to present. Blood was obtained from groups 2) and 3) while DNA from normal tissue was obtained from group 1). Data are available on age at diagnosis, race, pretreatment prostate-specific antigen (PSA) values, clinical pathology values, and family history. The control subjects were men undergoing disease screening and were not thought to have prostate cancer on the basis of a physical exam and a serum PSA value below 4 ng/ml. Screenings were performed at the Johns Hopkins Applied Physics Lab, at Bethlehem Steel in Baltimore, and at local African American churches in East Baltimore [7]. A total of 368 African American cases and 172 controls contributed to stage 1.

#### The Los Angeles Study of Aggressive Prostate Cancer (LAAPC)

The LAAPC is a population-based case-control study of aggressive prostate among African Americans in Los Angeles County [37]. Cases were identified through the Los Angeles County Cancer Surveillance Program rapid case ascertainment system and eligible cases included African American men diagnosed with a first primary prostate cancer between January 1, 1999 and December 31, 2003. Eligible cases also had either tumor extension outside the prostate, metastatic prostate cancer in sites other than prostate, or needle biopsy of the prostate with Gleason grade 8 or higher, or Gleason grade 7 and tumor in more than 2/3 of the biopsy cores. Controls were identified by a neighborhood walk algorithm and were men never diagnosed with prostate cancer, and were frequency matched to cases on age (±5 years). For this study, genomic DNA was included for 296 cases and 140 controls. We also included an additional 163 African American controls from the MEC that were frequency matched to cases on age.

#### Prostate Cancer Genetics Study (CaP Genes)

The African American component of this study population comprised 160 men: 75 cases diagnosed with more aggressive prostate cancer and 85 age-matched controls [38]. All subjects were recruited and frequency-matched on the major medical institutions in Cleveland, Ohio (i.e., the Cleveland Clinic, University Hospitals of Cleveland, and their affiliates) between 2001 and 2004. The cases were newly diagnosed with histologically confirmed disease: Gleason score 7; tumor stage T2c; or a prostate-specific antigen level >10 ng/ml at diagnosis. Controls were men without a prostate cancer diagnosis who underwent standard annual medical examinations at the collaborating medical institutions.

#### Case-Control Study of Prostate Cancer among African Americans in Washington, DC (DCPC)

Unrelated men self-described as African American were recruited for several case-control studies on genetic risk factors for prostate cancer between the years 2001 and 2005 from the Division of Urology at Howard University Hospital (HUH) in Washington, DC. Control subjects unrelated to the cases and matched for age (±5 years) were also ascertained from the prostate cancer screening population of the Division of Urology at HUH [24]. These studies included 292 cases and 359 controls.

#### King County (Washington) Prostate Cancer Studies (KCPCS)

The study population consists of participants from one of two population-based case-control studies among residents of King County, Washington [39], [40]. Incident Caucasian and African American cases with histologically confirmed prostate cancer were ascertained from the Seattle-Puget Sound SEER cancer registry during two time periods, 1993–1996 and 2002–2005. Age-matched (5-year age groups) controls were men without a self-reported history of being diagnosed with prostate cancer and were identified using one-step random digit telephone dialing. Controls were ascertained during the same time periods as the cases. A total of 145 incident African American cases and 81 African American controls were included from these studies.

#### The Gene-Environment Interaction in Prostate Cancer Study (GECAP)

The Henry Ford Health System (HFHS) recruited cases diagnosed with adenocarcinoma of the prostate of Caucasian or African American race, less than 75 years of age, and living in the metropolitan Detroit tri-county area [41]. Controls were randomly selected from the same HFHS population base from which cases were drawn. The control sample was frequency matched at a ratio of 3 enrolled cases to 1 control based on race and five-year age stratum. In total, 637 cases and 244 controls were enrolled between January 2002 and December 2004. Of study enrollees, DNA for 234 African Americans cases and 92 controls were included in stage 1 of the scan.

### Genotyping

Genotyping of 7,123 samples from these studies (3,621 cases and 3,502 controls) was conducted using the Illumina Infinium 1 M-Duo bead array at the University of Southern California and the NCI Genotyping Core Facility (PLCO study). Following genotyping samples were removed based on the following exclusion criteria: 1) unknown replicates across studies (n = 24, none within studies); 2) call rates <95% (n = 126); 3) samples with >10% mean heterozygosity on the X chromosome and/or <10% mean intensity on the Y chromosome - we inferred 3 samples to be XX and 6 to be XXY; 4) ancestry outliers (n = 108, discussed below), and; 5) samples that were related (n = 141, discussed below). To assess genotyping reproducibility we included 158 replicate samples; the average concordance rate was 99.99% (≥99.3% for all pairs). Starting with 1,153,397 SNPs, we removed SNPs with <95% call rate, MAFs<1%, or >1 QC mismatch based on sample replicates (n = 105,411). The analysis included 1,047,986 SNPs among 3,425 cases and 3,290 controls.

### Statistical Analysis

#### Relatedness inference

We used PLINK to calculate the probabilities of sharing 0, 1, and 2 alleles (Z = Z0, Z1, Z2) across all possible pairs of samples to determine individuals who were likely to be related to others within and across studies. We identified 167 pairs of related subjects (MZ twin, parent-offspring pairs, full and half-sibling pairs), based on the values of their observed probability vector Z being within 1 SD of the expected values of Z for their respective relationship. The criterion for removal was such that individuals that were connected with a higher number of pairs were chosen for removal. In all other cases, one of the two members was randomly selected for removal. A total of 141 subjects were removed.

#### Global ancestry estimation

The EIGENSTRAT software was used to calculate eigenvectors that explained genetic differences in ancestry among samples in the study [42]. The program included data from both HapMap Phase 3 populations and our study, so that comparisons to reference populations of known ethnicity could be made. An individual was subject to filtering from the analysis if his value along eigenvector 1 or 2 was outside of 4 SDs of the mean of each respective eigenvector. We identified 108 individuals who met this criterion. Eigenvector 1 was highly correlated (ρ = 0.997, p<1×10^−16^) with percentage of European ancestry, estimated in HAPMIX [43]. Together the top 10 eigenvectors (used in the analysis) explain 21% of the global genetic variability among subjects.

#### Local ancestry estimation

At each locus and for each participant, local ancestry was defined as the estimated number of European chromosomes (continuous between 0–2) carried by the participant, estimated via the HAPMIX program [43]. To summarize local ancestry at each region, for each individual we averaged across all local ancestry estimates that were within the start and end points of the region (Table S5). We used this average value as an additional covariate in the risk analyses.

#### SNP imputation

In order to generate a dataset suitable for fine-mapping, we carried out genome-wide imputation using the software MACH [44]. Phased haplotype data from the founders of the CEU (CEPH) and YRI (Yoruba) HapMap Phase 2 samples were used to infer LD patterns in order to impute ungenotyped markers. The Rsq metric, defined as the observed variance divided by the expected variance, provides a measure of the quality of the imputation at any SNP, and was used as a threshold in determining which SNPs to filter from analysis (Rsq<0.3). Of the 1,539,328 common SNPs (MAF≥0.05) in the YRI population in HapMap Phase 2, we could impute 1,392,294 (90%) with Rsq≥0.8. For all imputed SNPs presented in the Results and Tables reported herein, the average Rsq was 0.92 (estimated in MACH).

#### Association testing

For each typed and imputed SNP, odds ratios (OR) and 95% confidence intervals (95% CI) were estimated using unconditional logistic regression adjusting for age at diagnosis (or age at the reference date for controls), study, the first 10 eigenvalues and local ancestry. For each SNP, we tested for allele dosage effects through a 1 d.f. Wald chi-square trend test.

We fine-mapped each risk locus in search of 1) a better marker of the index signal in African Americans, and; 2) a novel signal that is independent of the index signal. These analyses included SNPs (genotyped and imputed) spanning 250 kb upstream and 250 kb downstream of each index signal. If the index signal was contained within an LD block (based on the D^′^ statistic) of >250 kb, then the region was extended to include the entire region of LD. Stepwise regression was performed by region to select the most informative risk variants as discussed below, in models adjusted for age, study, global ancestry (the 1^st^ eigenvector) and local ancestry. In the stepwise regression we preserved the original sample size by using the mean genotype of typed subjects in place of “no-calls” for SNPs with <100% genotyping completion rate.

Within each known risk locus, it is expected that markers that are associated with risk in African Americans will be correlated with the index signal reported in Europeans. Thus, we identified and tested SNPs that are correlated (r^2^>0.2) with the index signals in Europeans in HapMap (CEU population). Because these variants are not independent and there is a high prior probability that signals exist among such variants, we applied a lenient criteria for keeping them in the stepwise regression. The average number of tags to capture (r^2^>0.8) these SNPs in each region was used as a correction factor, as they define the number of independent tests (p<0.004). For all of the remaining markers that were not correlated with the index signal (in Europeans), we applied a more stringent α level for defining statistical significance. In each risk region, we determined the number of tag SNPs needed to capture all common alleles (MAF>0.05, with r^2^>0.8) in the YRI population in Phase 2 HapMap using single and multi-marker tests. An α of 0.05/the total number of tags was applied to assess statistical significance for any putative novel, independent signal in each region (p<5.6×10^−6^). For the correlated SNPs we had 80% power to detect an OR of 1.17 per copy for a 20% risk allele, whereas for the novel SNPs the detectable OR for such an allele increased to 1.26 per copy. A similar stepwise analysis was also performed at 8q24 (127.8–129.0 Mb) for SNPs with nominal p-values<0.05, keeping SNPs if p<0.001 in the multivariate model. This choice of p-value reflects a balance between the need to correct for multiple comparisons and the prior knowledge that this region harbors multiple independent risk alleles for prostate cancer. For SNPs in the 8q24 region we had 80% power to detect an OR of 1.19 per copy for a 20% risk allele. We tested heterogeneity of effect by study for all 76 SNPs presented in Table 1 and Table 2 and we observed 5 significant associations (p<0.05, 3.6 expected) and only 1 at p<0.01 (rs7000448 at 8q24, p = 0.004).

#### Risk modeling

We modeled the cumulative genetic risk of prostate cancer using the risk variants reported in previous GWAS (total = 40). For regions outside of 8q24 with multiple correlated variants, we selected the SNP with the largest OR in African Americans. At 8q24 we used the seven variants reported in Al Olama et al. [1]. We compared the results to a model of the SNPs found to be significantly associated with risk in African Americans, which included the index signals if nominally associated with risk in African Americans (p≤0.05) as well as SNPs identified from the stepwise procedures at all loci including 8q24 (total = 27). More specifically, in each case we summed the number of risk alleles for each individual and estimated the odds ratio per allele for this aggregate unweighted allele count variable as an approximate risk score appropriate for unlinked variants with independent effects of approximately the same magnitude for each allele. For individuals missing genotypes for a given SNP, we assigned the average number of risk alleles (2× risk allele frequency) to replace the missing value for that SNP. To address the independence assumption, we compared the betas for each SNP with the betas obtained when all SNPs were included in the same model. We found remarkable consistency in the betas, which supports their associations as being independent (Table S9). We also stratified the risk score analysis by first-degree family history of prostate cancer. We tested for differences in the effect of the risk score by disease severity (advanced disease defined as Gleason 8–10 and/or non-localized stage vs non-advanced disease defined as Gleason≤7 and localized stage).

#### Heritability explained by the score

We estimated crudely how much of the familial risk of prostate cancer is explained by the known risk alleles as summarized in the improved risk score. In this study, a first-degree family history of prostate cancer is associated with a relative risk of 1.55 (95% CI, 1.32–1.81). Making the simplifying assumption that all risk alleles are inherited independently then the correlation between the risk allele count for two first-degree relatives will be equal to 0.5 (i.e. will equal 1/2 the probability of sharing one allele IBD+the probability of sharing two alleles IBD). Making the further assumption that the number of risk alleles is distributed as approximately normal with mean = 30.66 and standard deviation 3.07 alleles in the population (estimated among African American controls) and that in cases the mean is 32.13 alleles with roughly the same standard deviation (3.08), we can approximate the mean number of alleles in individuals of unknown prostate cancer status, but each of whom has a single first-degree relative (brother or father) with the disease as 30.66(1–0.5^2^)+32.13(0.5^2^) = 31.03. Since this is just 0.37 more alleles than is expected in the control population overall we see that the relative odds of prostate cancer for a man with a brother or father with prostate cancer is only exp(log(1.17)*0.37) = 1.06 higher than an unselected subject (i.e. one not selected on the basis of disease in a first-degree relative). Compared to the approximately 1.55-fold increase in relative risk, this risk score may only explain ∼11% [(1.06−1)/(1.55−1)×100%] of risk in first-degree relatives of cases, which indicates that many more alleles are required to explain familial aggregation in the African American population.

## Supporting Information

Figure S1 Linkage disequilibrium plots of prostate cancer risk regions in the GWAS population and Yorubans (YRI).
(2.61 MB DOCX)

Figure S2 Pairwise correlation (r^2^) of known risk variants at 8q24 in African Americans estimated in 941 African Americans in the MEC.
(0.04 MB DOCX)

Table S1 Descriptive characteristics of the 11 studies included in stage 1 of the GWAS of prostate cancer in African Americans.
(0.02 MB DOCX)

Table S2 Power to detect associations with the known risk variants in African American.
(0.05 MB DOCX)

Table S3 The association of local ancestry surrounding the index signal(s) at each risk locus and prostate cancer risk.
(0.02 MB DOCX)

Table S4 Associations with established risk variants for prostate cancer (3,425 cases, 3,290 controls) adjusted for global ancestry.
(0.02 MB DOCX)

Table S5 Information about the 27 regions fine-mapped (not including 8q24).
(0.03 MB DOCX)

Table S6 Results of the stepwise procedure in each risk region (not including 8q24).
(0.02 MB DOCX)

Table S7 Associations by genotype class for SNPs in known prostate cancer risk regions that were found to be nominally associated with risk in African Americans.
(0.02 MB DOCX)

Table S8 Correlations (r^2^) between risk markers in African Africans and known risk variants at 8q24.
(0.02 MB DOCX)

Table S9 Independence of markers utilized in risk modeling.
(0.02 MB DOCX)

## References

[1] Al Olama AA, Kote-Jarai Z, Giles GG, Guy M, Morrison J (2009). Multiple loci on 8q24 associated with prostate cancer susceptibility.. Nat Genet.

[2] Amundadottir LT, Sulem P, Gudmundsson J, Helgason A, Baker A (2006). A common variant associated with prostate cancer in European and African populations.. Nat Genet.

[3] Eeles RA, Kote-Jarai Z, Al Olama AA, Giles GG, Guy M (2009). Identification of seven new prostate cancer susceptibility loci through a genome-wide association study.. Nat Genet.

[4] Eeles RA, Kote-Jarai Z, Giles GG, Olama AA, Guy M (2008). Multiple newly identified loci associated with prostate cancer susceptibility.. Nat Genet.

[5] Freedman ML, Haiman CA, Patterson N, McDonald GJ, Tandon A (2006). Admixture mapping identifies 8q24 as a prostate cancer risk locus in African-American men.. Proc Natl Acad Sci U S A.

[6] Gudmundsson J, Sulem P, Gudbjartsson DF, Blondal T, Gylfason A (2009). Genome-wide association and replication studies identify four variants associated with prostate cancer susceptibility.. Nat Genet.

[7] Gudmundsson J, Sulem P, Manolescu A, Amundadottir LT, Gudbjartsson D (2007). Genome-wide association study identifies a second prostate cancer susceptibility variant at 8q24.. Nat Genet.

[8] Gudmundsson J, Sulem P, Rafnar T, Bergthorsson JT, Manolescu A (2008). Common sequence variants on 2p15 and Xp11.22 confer susceptibility to prostate cancer.. Nat Genet.

[9] Gudmundsson J, Sulem P, Steinthorsdottir V, Bergthorsson JT, Thorleifsson G (2007). Two variants on chromosome 17 confer prostate cancer risk, and the one in TCF2 protects against type 2 diabetes.. Nat Genet.

[10] Haiman CA, Patterson N, Freedman ML, Myers SR, Pike MC (2007). Multiple regions within 8q24 independently affect risk for prostate cancer.. Nat Genet.

[11] Takata R, Akamatsu S, Kubo M, Takahashi A, Hosono N (2010). Genome-wide association study identifies five new susceptibility loci for prostate cancer in the Japanese population.. Nat Genet.

[12] Thomas G, Jacobs KB, Yeager M, Kraft P, Wacholder S (2008). Multiple loci identified in a genome-wide association study of prostate cancer.. Nat Genet.

[13] Yeager M, Chatterjee N, Ciampa J, Jacobs KB, Gonzalez-Bosquet J (2009). Identification of a new prostate cancer susceptibility locus on chromosome 8q24.. Nat Genet.

[14] Yeager M, Orr N, Hayes RB, Jacobs KB, Kraft P (2007). Genome-wide association study of prostate cancer identifies a second risk locus at 8q24.. Nat Genet.

[15] Gail MH (2009). Value of adding single-nucleotide polymorphism genotypes to a breast cancer risk model.. J Natl Cancer Inst.

[16] Wacholder S, Hartge P, Prentice R, Garcia-Closas M, Feigelson HS (2010). Performance of common genetic variants in breast-cancer risk models.. N Engl J Med.

[17] Sun J, Zheng SL, Wiklund F, Isaacs SD, Purcell LD (2008). Evidence for two independent prostate cancer risk-associated loci in the HNF1B gene at 17q12.. Nat Genet.

[18] Zheng SL, Stevens VL, Wiklund F, Isaacs SD, Sun J (2009). Two independent prostate cancer risk-associated Loci at 11q13.. Cancer Epidemiol Biomarkers Prev.

[19] Waters KM, Le Marchand L, Kolonel LN, Monroe KR, Stram DO (2009). Generalizability of associations from prostate cancer genome-wide association studies in multiple populations.. Cancer Epidemiol Biomarkers Prev.

[20] Jia L, Landan G, Pomerantz M, Jaschek R, Herman P (2009). Functional enhancers at the gene-poor 8q24 cancer-linked locus.. PLoS Genet.

[21] Kote-Jarai Z, Easton DF, Stanford JL, Ostrander EA, Schleutker J (2008). Multiple novel prostate cancer predisposition loci confirmed by an international study: the PRACTICAL Consortium.. Cancer Epidemiol Biomarkers Prev.

[22] Rafnar T, Sulem P, Stacey SN, Geller F, Gudmundsson J (2009). Sequence variants at the TERT-CLPTM1L locus associate with many cancer types.. Nat Genet.

[23] Xu J, Zheng SL, Isaacs SD, Wiley KE, Wiklund F (2010). Inherited genetic variant predisposes to aggressive but not indolent prostate cancer.. Proc Natl Acad Sci U S A.

[24] Robbins C, Torres JB, Hooker S, Bonilla C, Hernandez W (2007). Confirmation study of prostate cancer risk variants at 8q24 in African Americans identifies a novel risk locus.. Genome Res.

[25] Crowther-Swanepoel D, Broderick P, Di Bernardo MC, Dobbins SE, Torres M (2010). Common variants at 2q37.3, 8q24.21, 15q21.3 and 16q24.1 influence chronic lymphocytic leukemia risk.. Nat Genet.

[26] Easton DF, Pooley KA, Dunning AM, Pharoah PD, Thompson D (2007). Genome-wide association study identifies novel breast cancer susceptibility loci.. Nature.

[27] Goode EL, Chenevix-Trench G, Song H, Ramus SJ, Notaridou M (2010). A genome-wide association study identifies susceptibility loci for ovarian cancer at 2q31 and 8q24.. Nat Genet.

[28] Kiemeney LA, Thorlacius S, Sulem P, Geller F, Aben KK (2008). Sequence variant on 8q24 confers susceptibility to urinary bladder cancer.. Nat Genet.

[29] Xiao R, Boehnke M (2009). Quantifying and correcting for the winner's curse in genetic association studies.. Genet Epidemiol.

[30] Chang BL, Spangler E, Gallagher S, Haiman CA, Henderson BE (2010). Validation of Genome-Wide Prostate Cancer Associations in Men of African Descent.. Cancer Epidemiol Biomarkers Prev.

[31] Xu J, Kibel AS, Hu JJ, Turner AR, Pruett K (2009). Prostate cancer risk associated loci in African Americans.. Cancer Epidemiol Biomarkers Prev.

[32] Kolonel LN, Henderson BE, Hankin JH, Nomura AM, Wilkens LR (2000). A multiethnic cohort in Hawaii and Los Angeles: baseline characteristics.. Am J Epidemiol.

[33] Signorello LB, Hargreaves MK, Steinwandel MD, Zheng W, Cai Q (2005). Southern community cohort study: establishing a cohort to investigate health disparities.. J Natl Med Assoc.

[34] Gohagan JK, Prorok PC, Hayes RB, Kramer BS (2000). The Prostate, Lung, Colorectal and Ovarian (PLCO) Cancer Screening Trial of the National Cancer Institute: history, organization, and status.. Control Clin Trials.

[35] Calle EE, Rodriguez C, Jacobs EJ, Almon ML, Chao A (2002). The American Cancer Society Cancer Prevention Study II Nutrition Cohort: rationale, study design, and baseline characteristics.. Cancer.

[36] Strom SS, Gu Y, Zhang H, Troncoso P, Babaian RJ (2004). Androgen receptor polymorphisms and risk of biochemical failure among prostatectomy patients.. Prostate.

[37] Ingles SA, Coetzee GA, Ross RK, Henderson BE, Kolonel LN (1998). Association of prostate cancer with vitamin D receptor haplotypes in African-Americans.. Cancer Res.

[38] Liu X, Plummer SJ, Nock NL, Casey G, Witte JS (2006). Nonsteroidal antiinflammatory drugs and decreased risk of advanced prostate cancer: modification by lymphotoxin alpha.. Am J Epidemiol.

[39] Agalliu I, Salinas CA, Hansten PD, Ostrander EA, Stanford JL (2008). Statin use and risk of prostate cancer: results from a population-based epidemiologic study.. Am J Epidemiol.

[40] Stanford JL, Wicklund KG, McKnight B, Daling JR, Brawer MK (1999). Vasectomy and risk of prostate cancer.. Cancer Epidemiol Biomarkers Prev.

[41] Rybicki BA, Neslund-Dudas C, Nock NL, Schultz LR, Eklund L (2006). Prostate cancer risk from occupational exposure to polycyclic aromatic hydrocarbons interacting with the GSTP1 Ile105Val polymorphism.. Cancer Detect Prev.

[42] Price AL, Patterson NJ, Plenge RM, Weinblatt ME, Shadick NA (2006). Principal components analysis corrects for stratification in genome-wide association studies.. Nat Genet.

[43] Price AL, Tandon A, Patterson N, Barnes KC, Rafaels N (2009). Sensitive detection of chromosomal segments of distinct ancestry in admixed populations.. PLoS Genet.

[44] Li Y, Willer C, Sanna S, Abecasis G (2009). Genotype imputation.. Annu Rev Genomics Hum Genet.

[45] Pruim RJ, Welch RP, Sanna S, Teslovich TM, Chines PS (2010). LocusZoom: regional visualization of genome-wide association scan results.. Bioinformatics.

