# Supplementary material for: Characterizing Genetic Risk at Known Prostate Cancer Susceptibility Loci in African Americans
Source: PLoS Genet. 2011 May 26;7(5):e1001387. doi: 10.1371/journal.pgen.1001387 (PMC3102736; doi:10.1371/journal.pgen.1001387)
Supplement: Figure S1 — Linkage disequilibrium plots of prostate cancer risk regions in the GWAS population and Yorubans (YRI). (2.61 MB DOCX) [file pgen.1001387.s001.docx]

**2p24, CHB+JPT HapMap Phase 2**

**
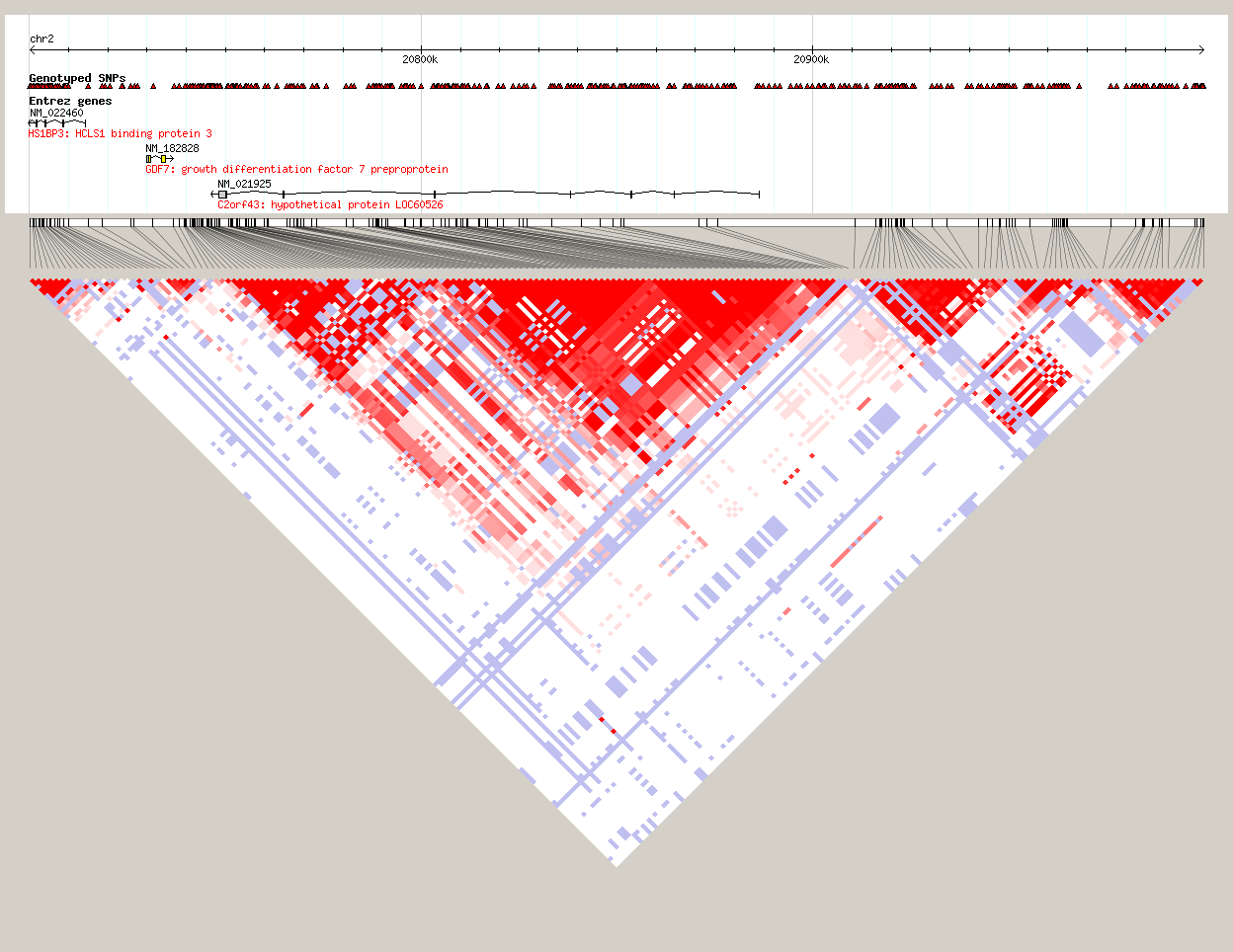
**

Signal in African Americans

Index Signal

**2p24, YRI HapMap Phase 2**

**
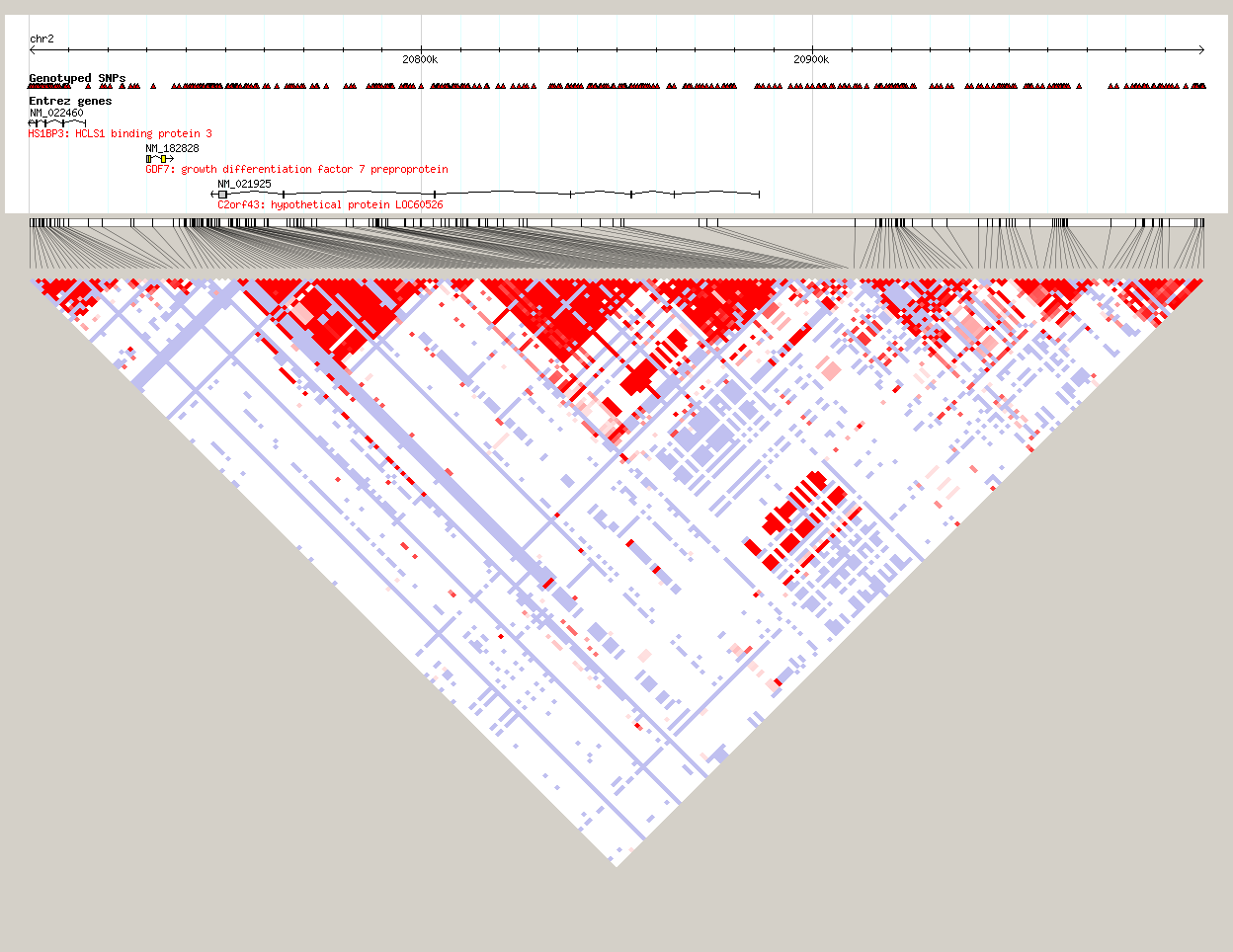
**

Signal in African Americans

Index Signal

Signal in African Americans

Index Signal

Index Signal

**2p15 CEU HapMap Phase 2**

**
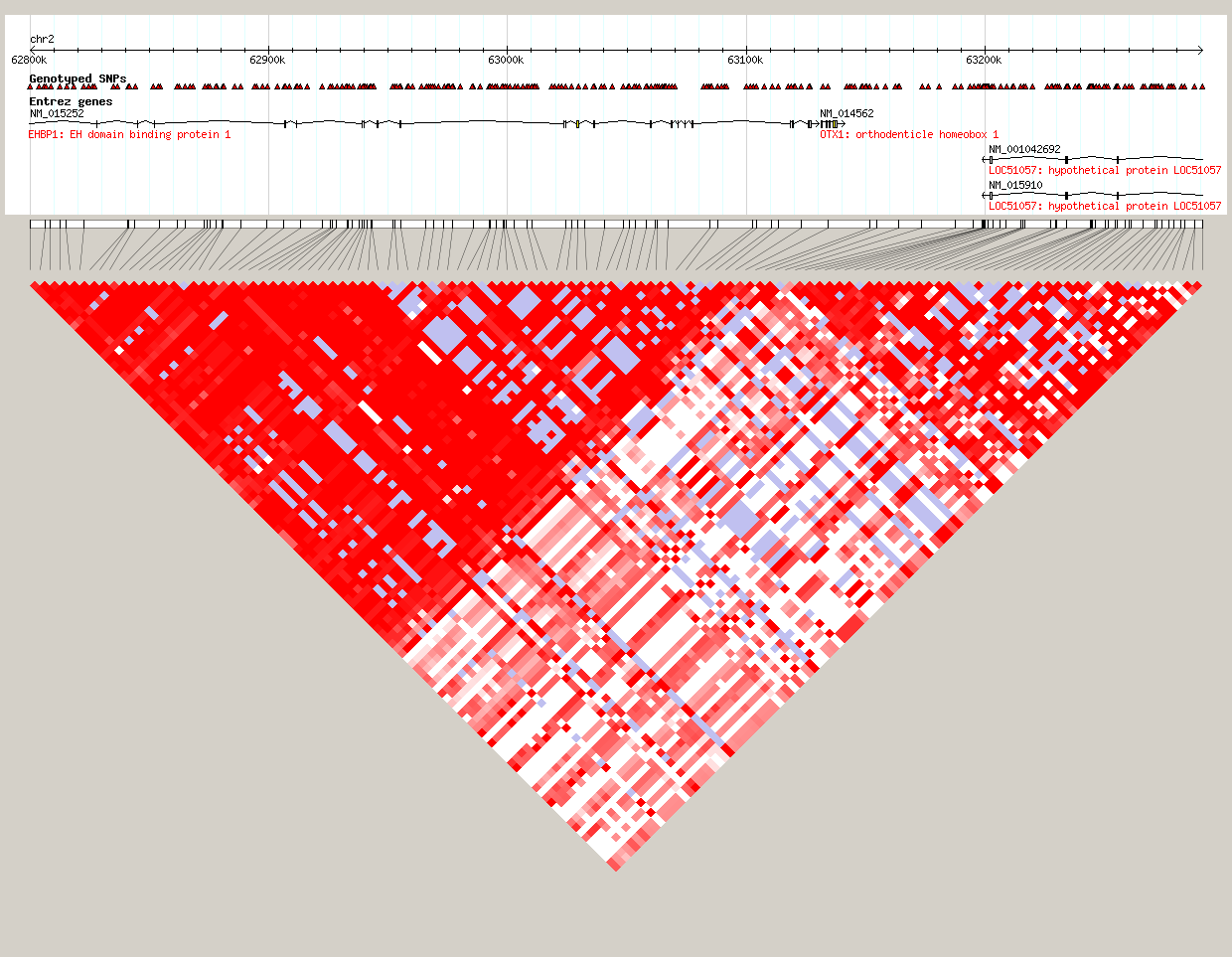
**

Signal in African Americans

Index Signal

**2p15 YRI HapMap Phase 2**

**
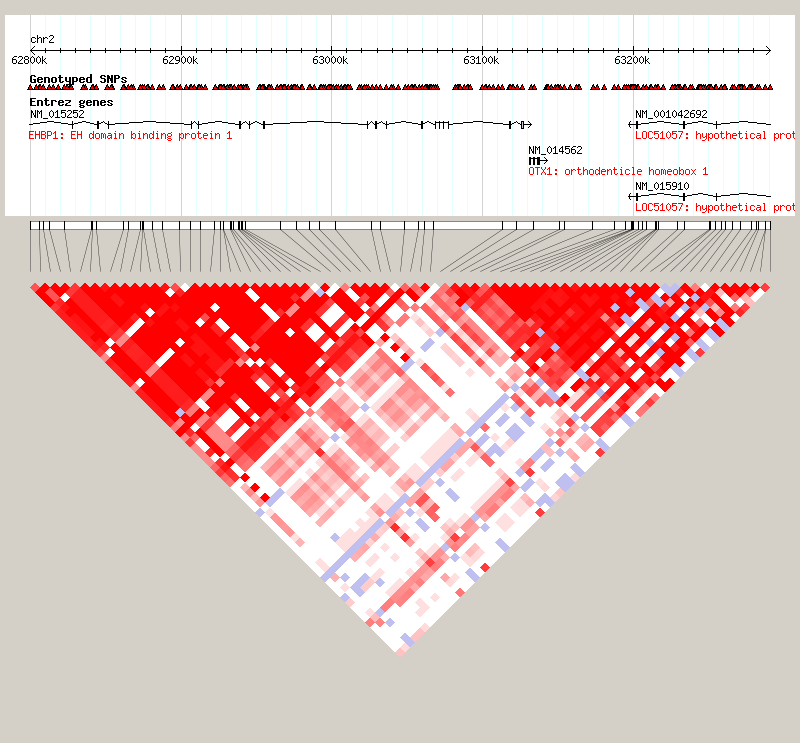
**

Signal in African Americans

Index Signal

**3q21, CEU HapMap Phase 2**

**
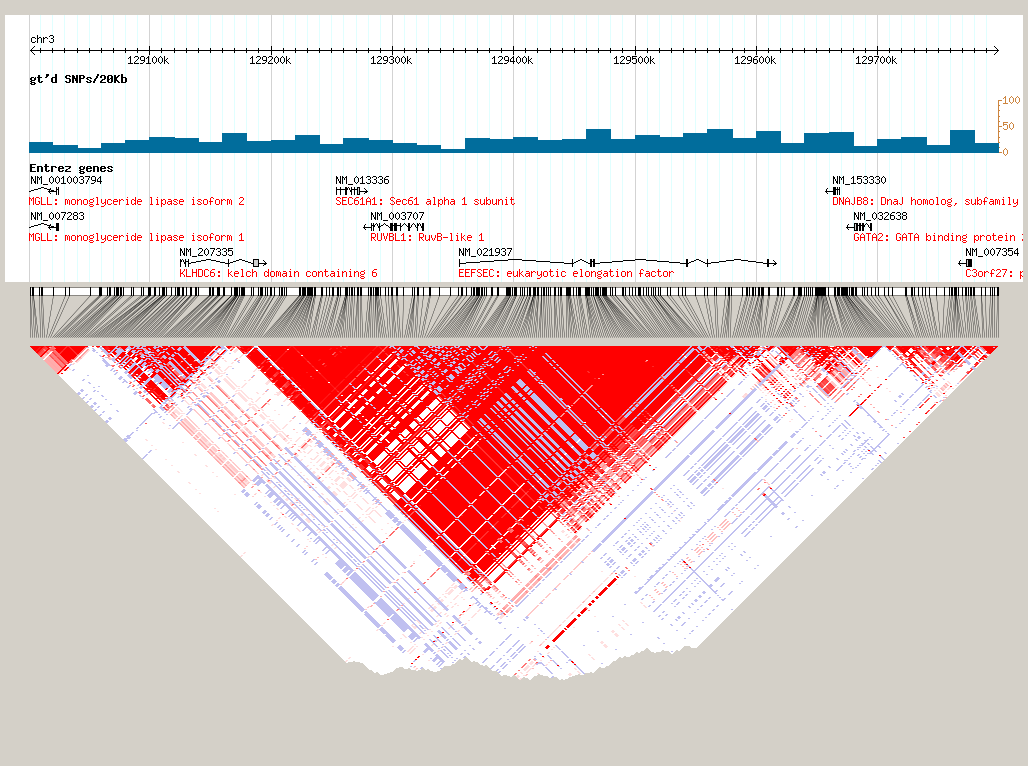
**

Index Signal

Signal in African Americans

**3q21, YRI HapMap Phase 2**

**
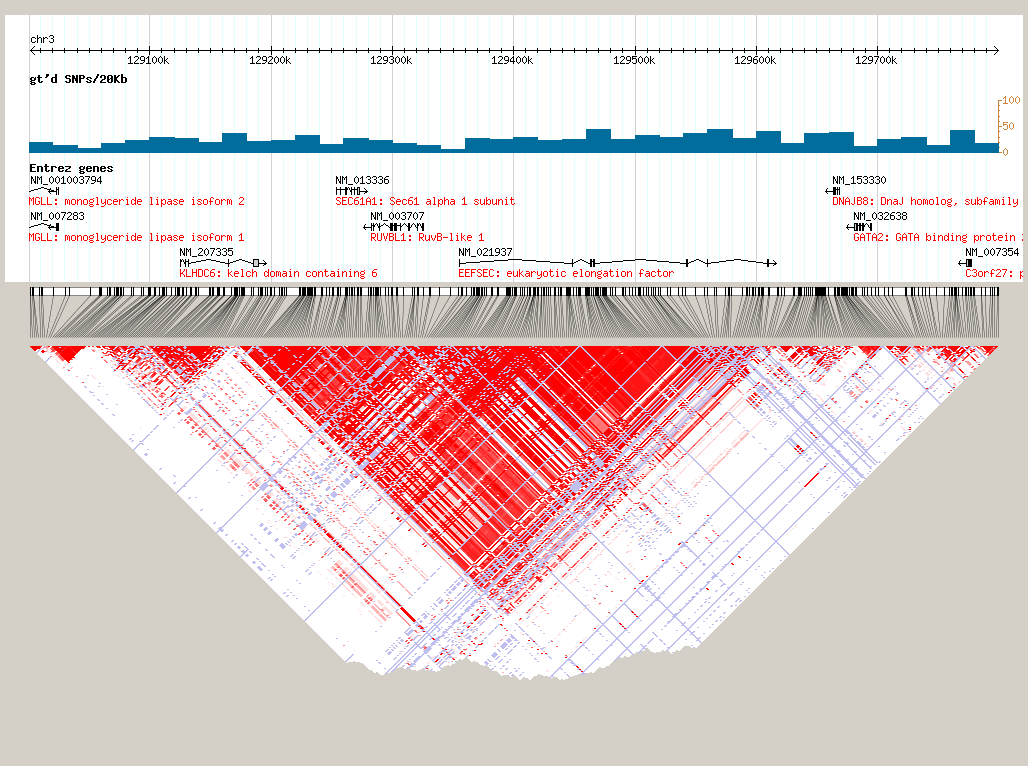
**

Signal in African Americans

Index Signal

Index Signal

Signal in African Americans

**6q22, CHB+JPT HapMap Phase 2**

**
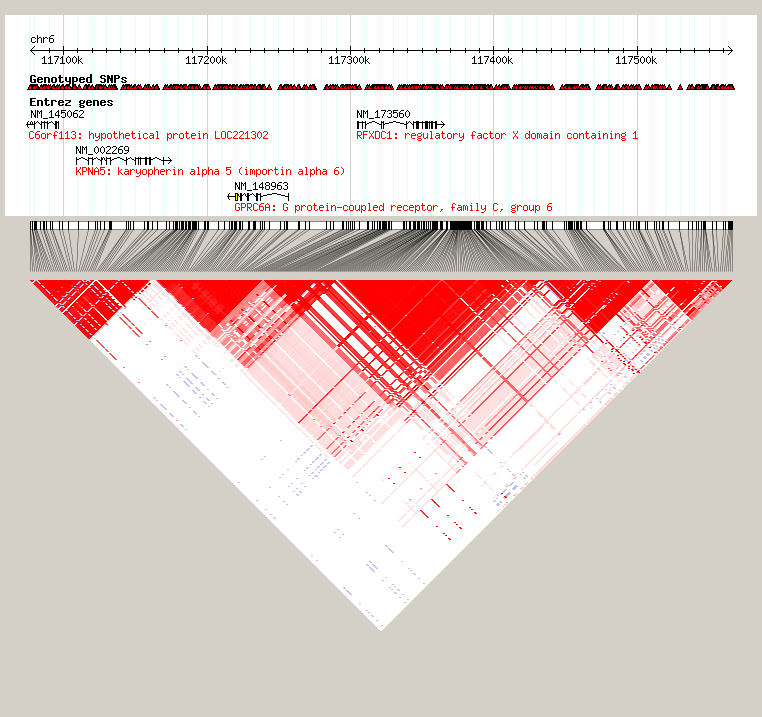
**

Signal in African Americans

Index Signal

**6q22, YRI HapMap Phase 2**

**
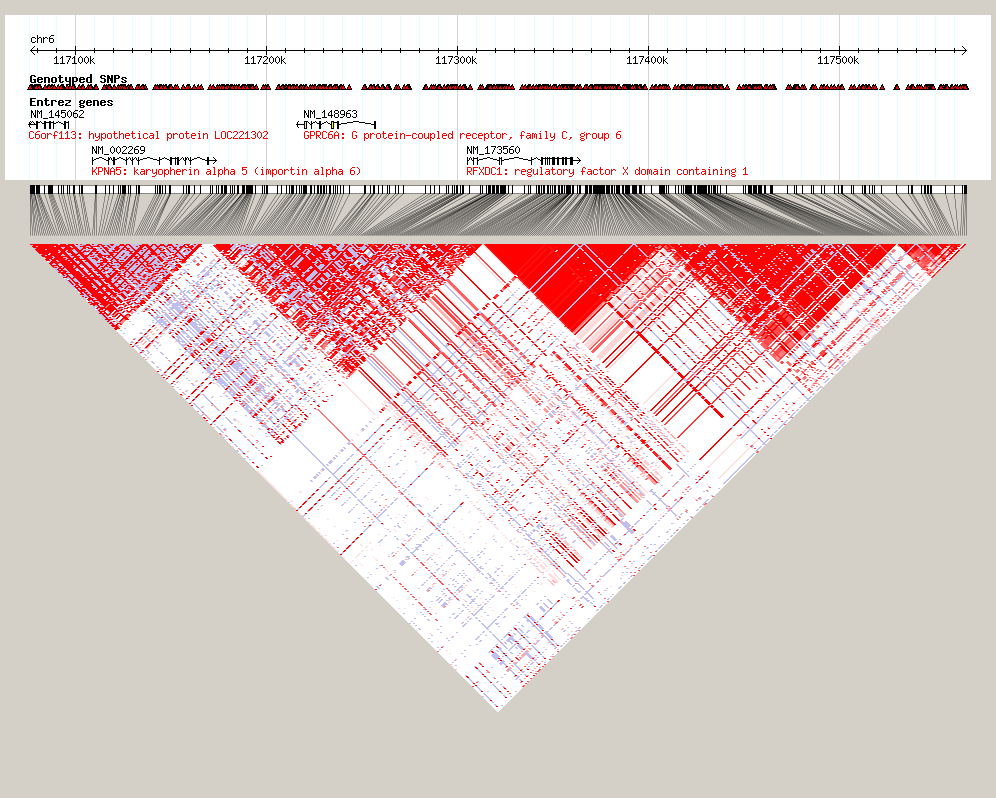
**

Signal in African Americans

Index Signal

**6q25, CEU HapMap Phase 2**

**
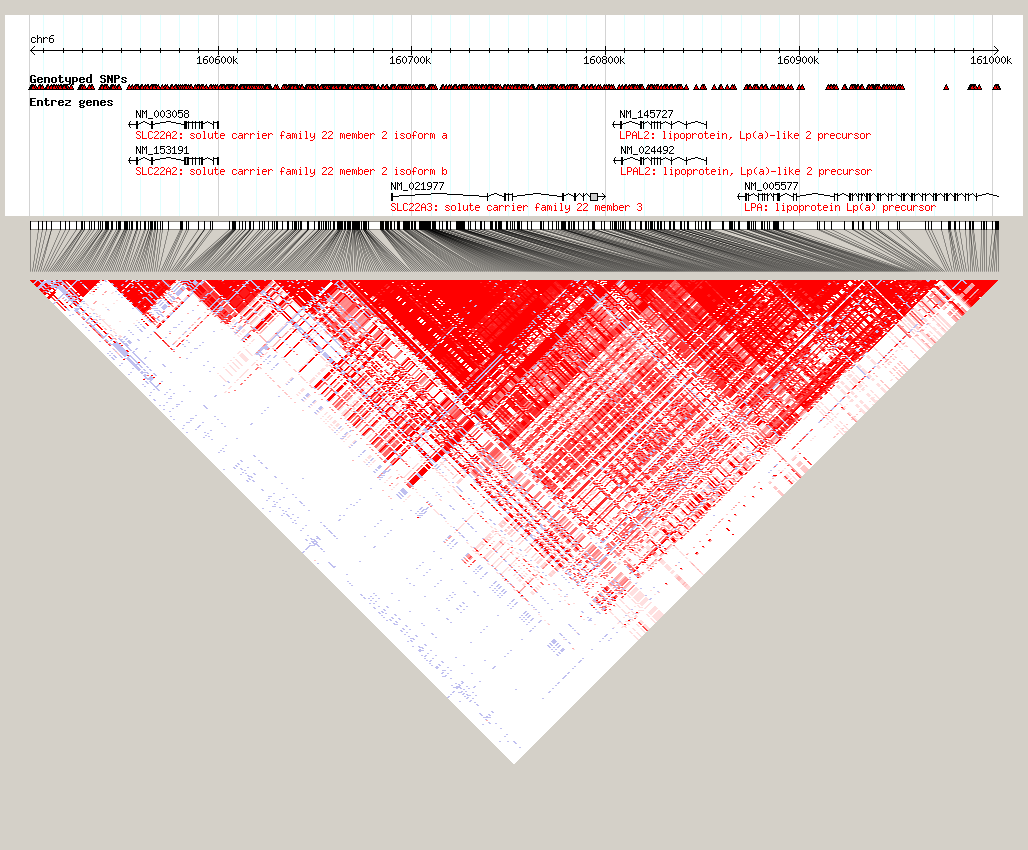
**

Signal in African Americans

Index Signal

**6q25, YRI HapMap Phase 2**

**
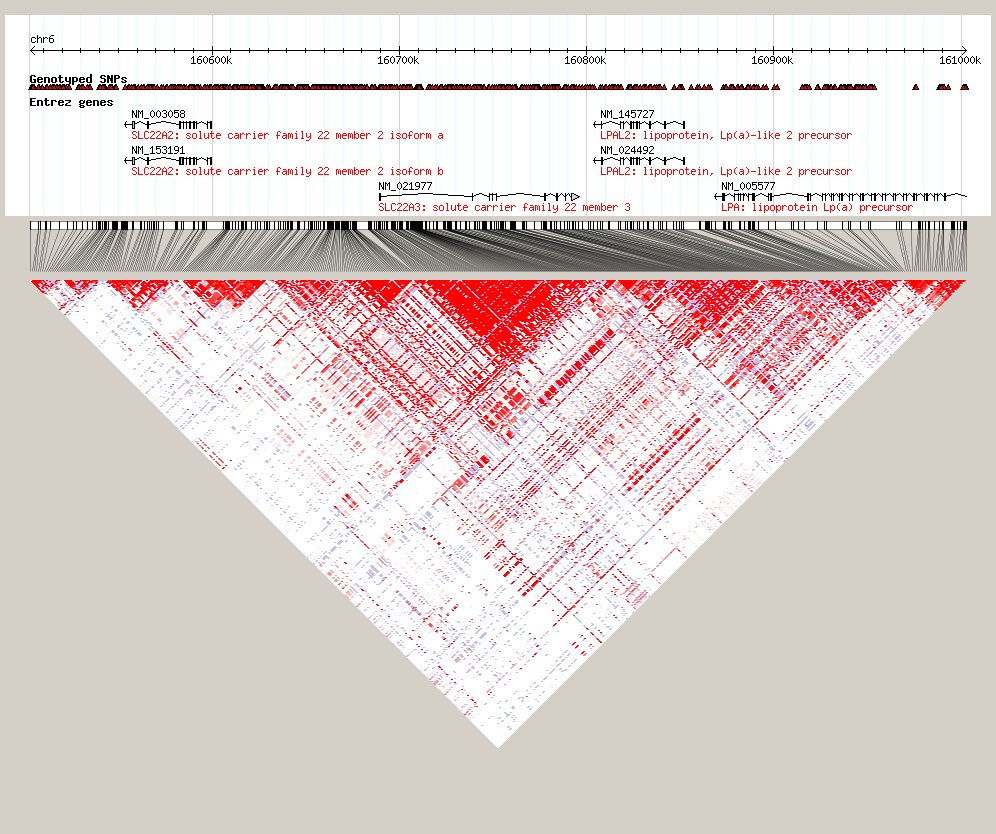
**

Signal in African Americans

Index Signal

**7p15, CEU HapMap Phase 2**

**
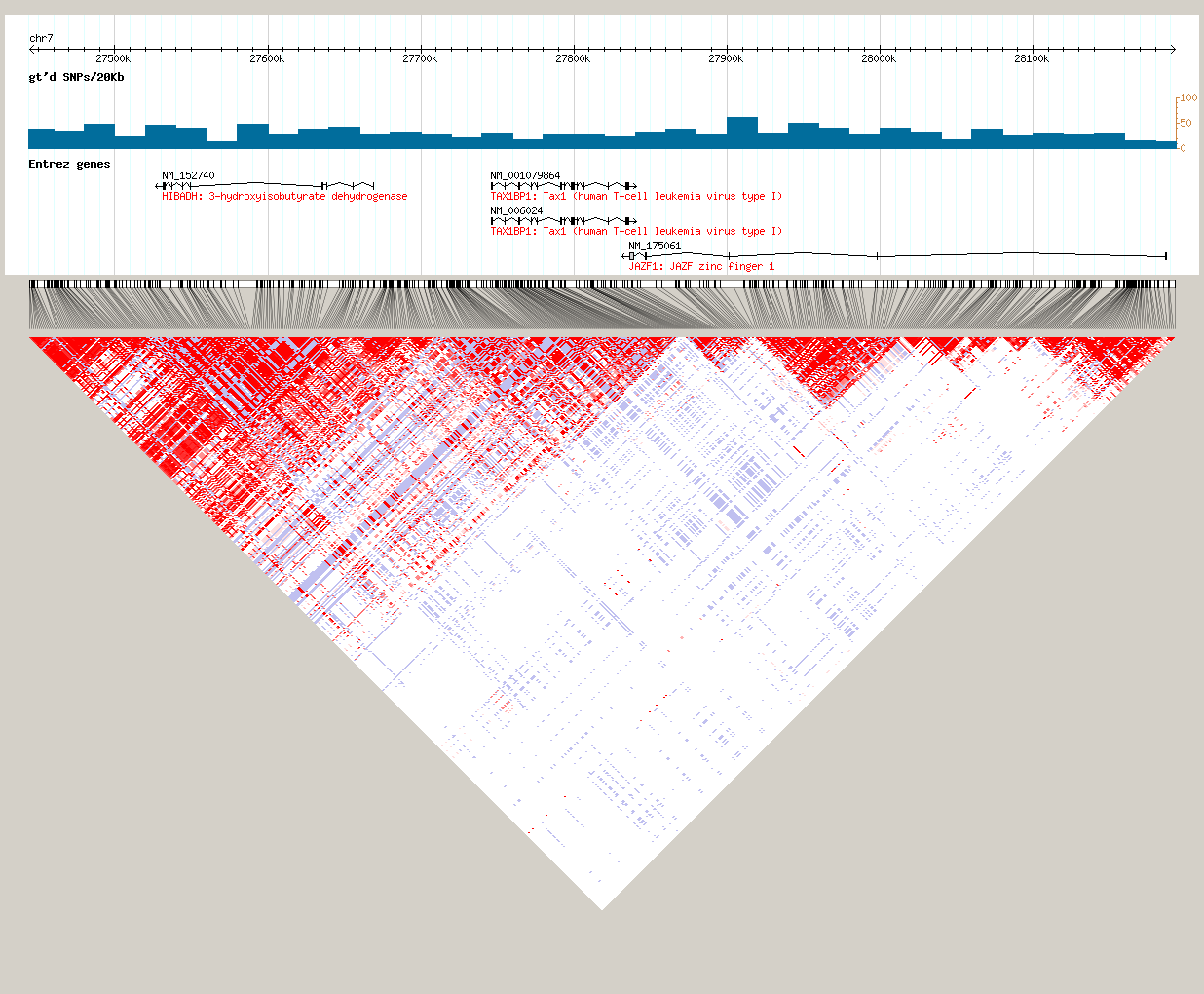
**

Index Signal

Signal in African Americans

Index Signal

**7p15, YRI HapMap Phase 2**

**
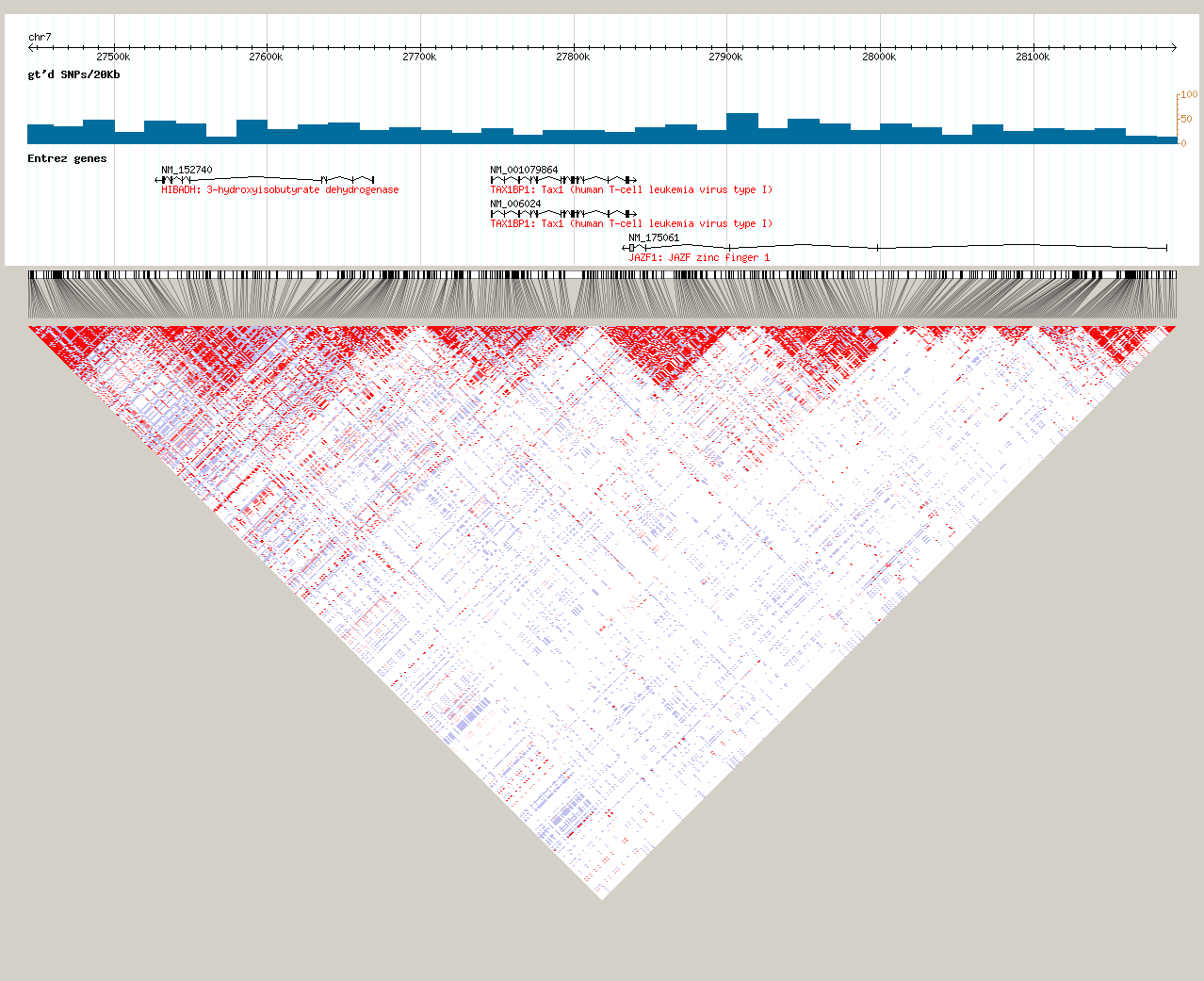
**

Signal in African Americans

Index Signal

**8p21, CEU HapMap Phase 2**

**
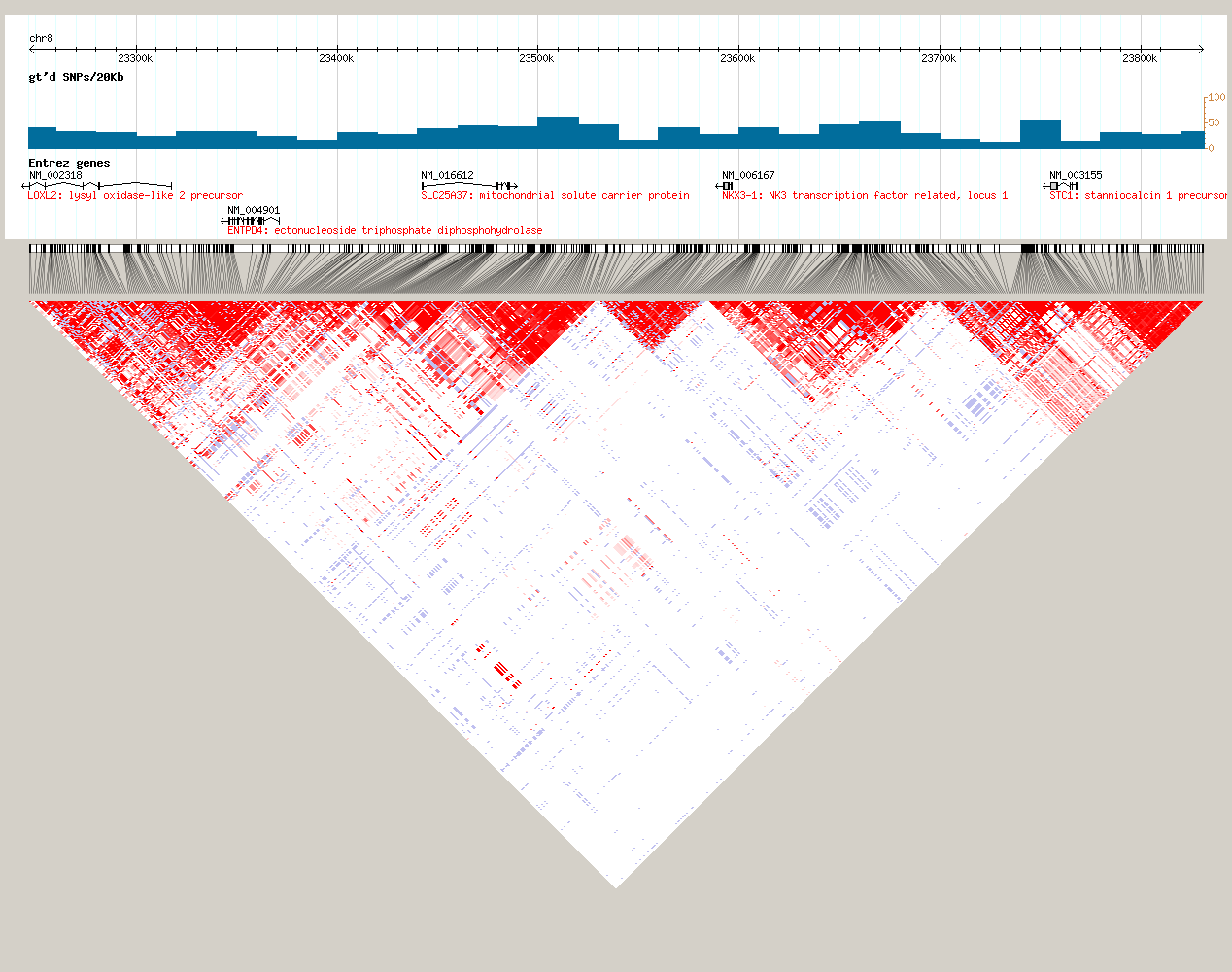
**

Signal in African Americans

Index Signal

**8p21, YRI HapMap Phase 2**

**
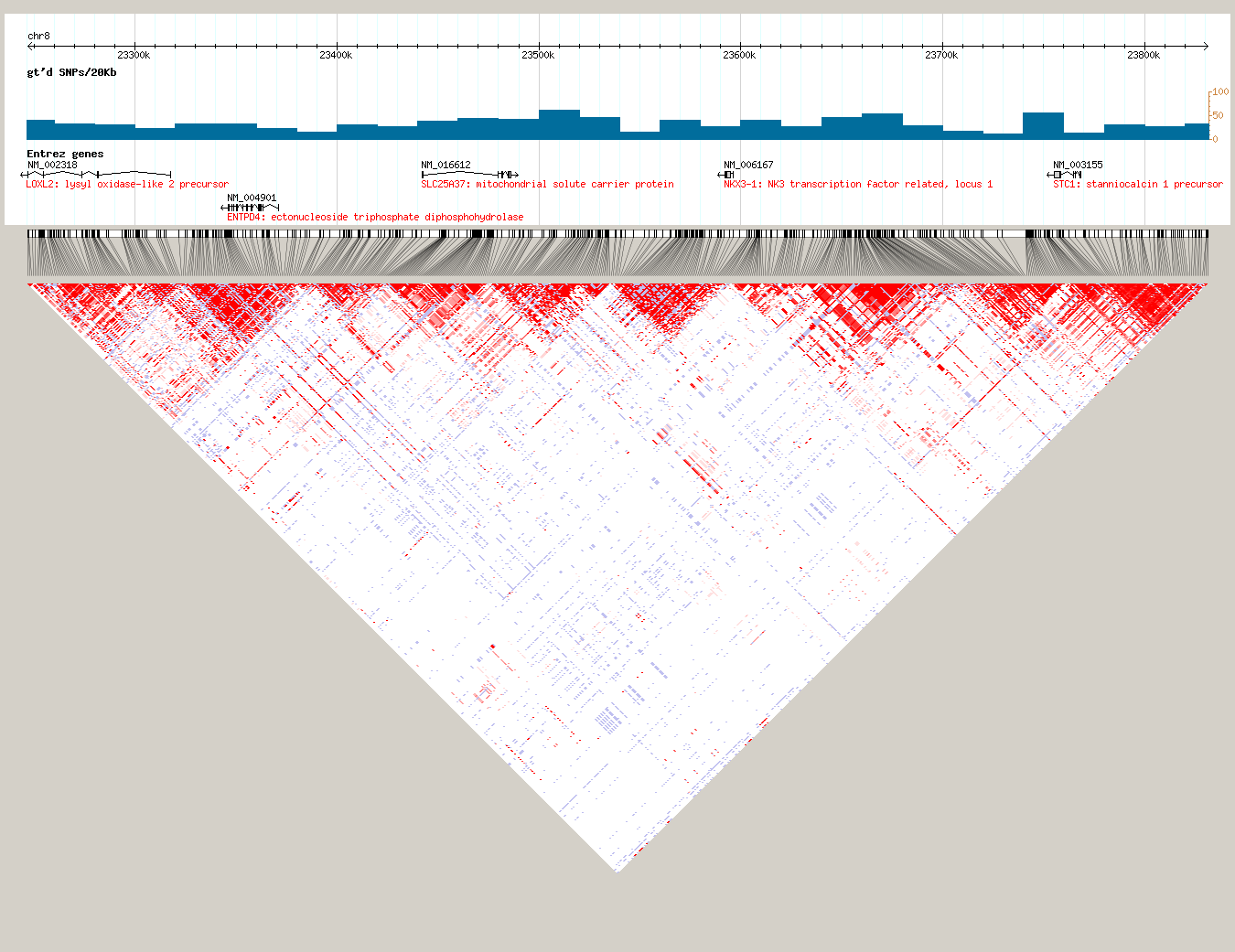
**

Signal in African Americans

Index Signal

**10q11, CEU HapMap Phase 2**

**
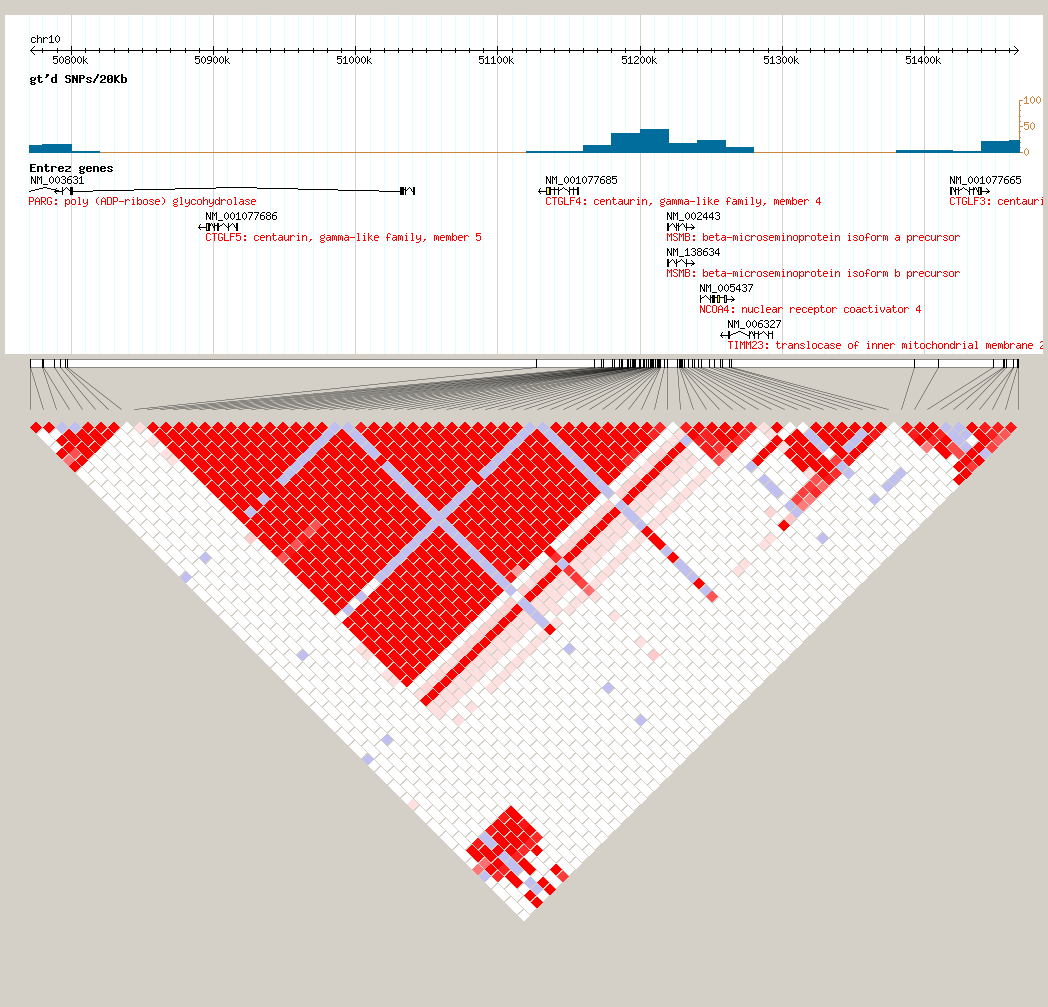
**

Signal in African Americans

Index Signal

**10q11, YRI HapMap Phase 2**

**
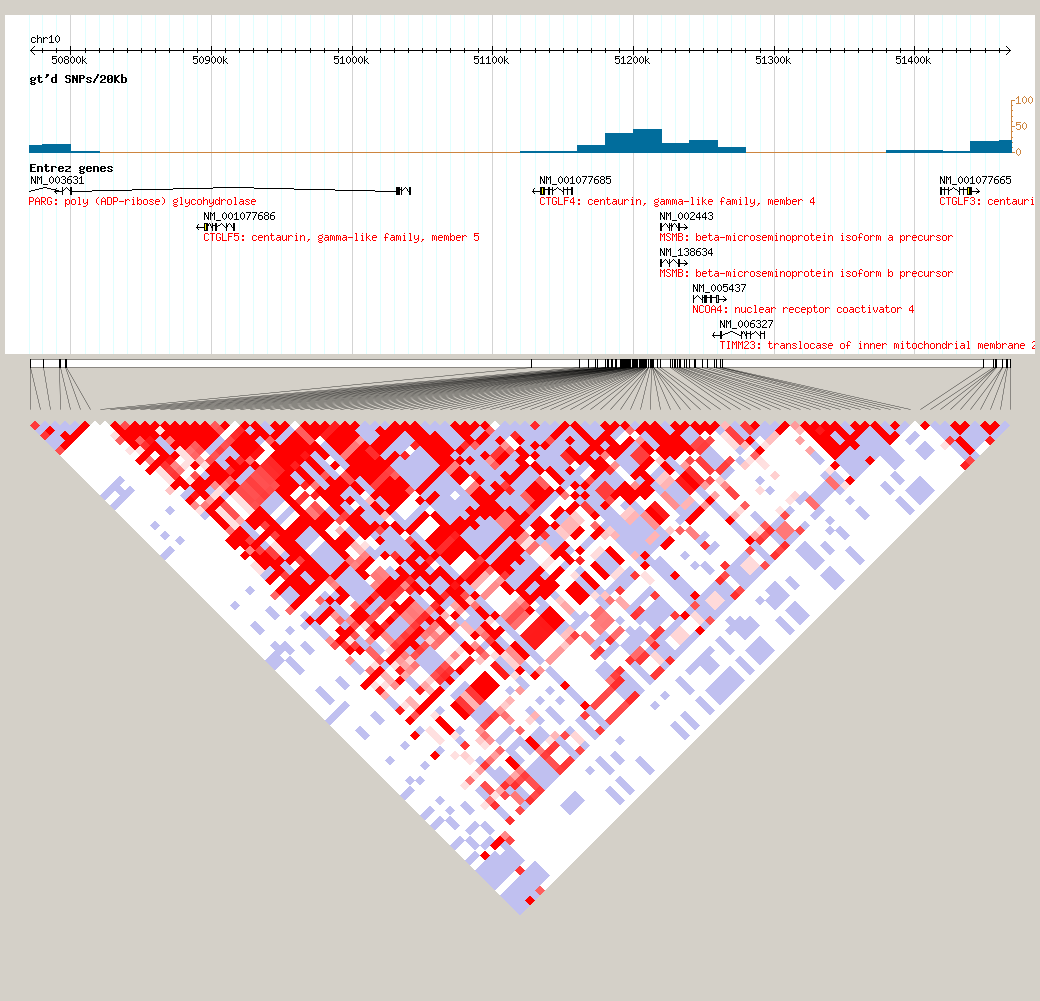
**

Index Signal

Signal in African Americans

**11q13, CEU HapMap Phase 2**

**
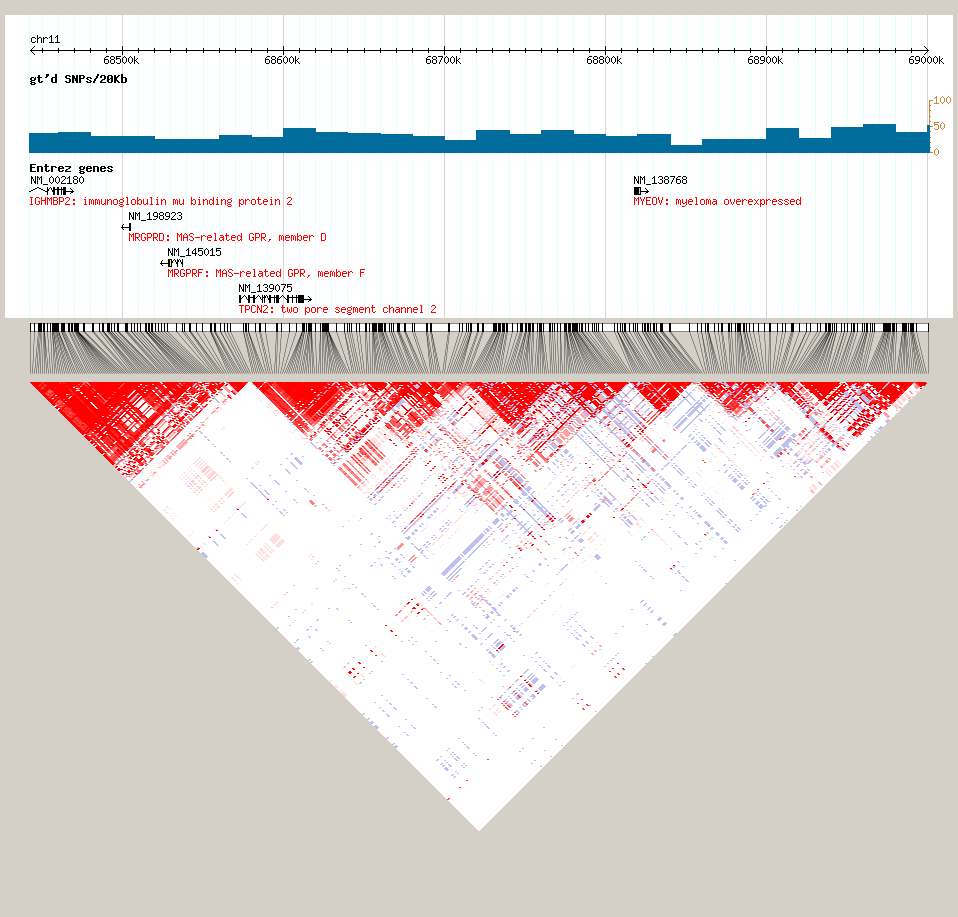
**

Signal in African Americans

Index Signals

**11q13, YRI HapMap Phase 2**

**
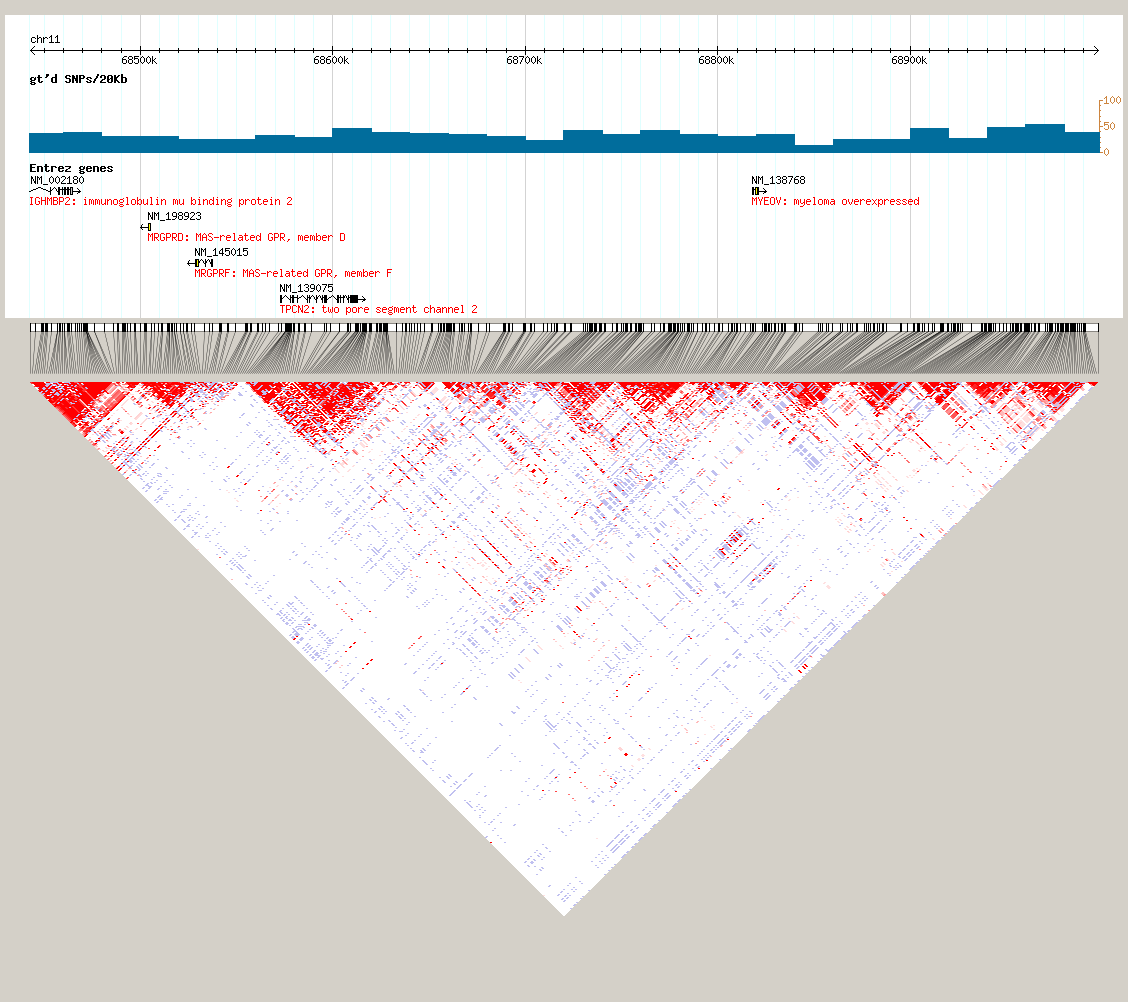
**

Signal in African Americans

Index Signals

**19q13, CEU HapMap Phase 2**

**
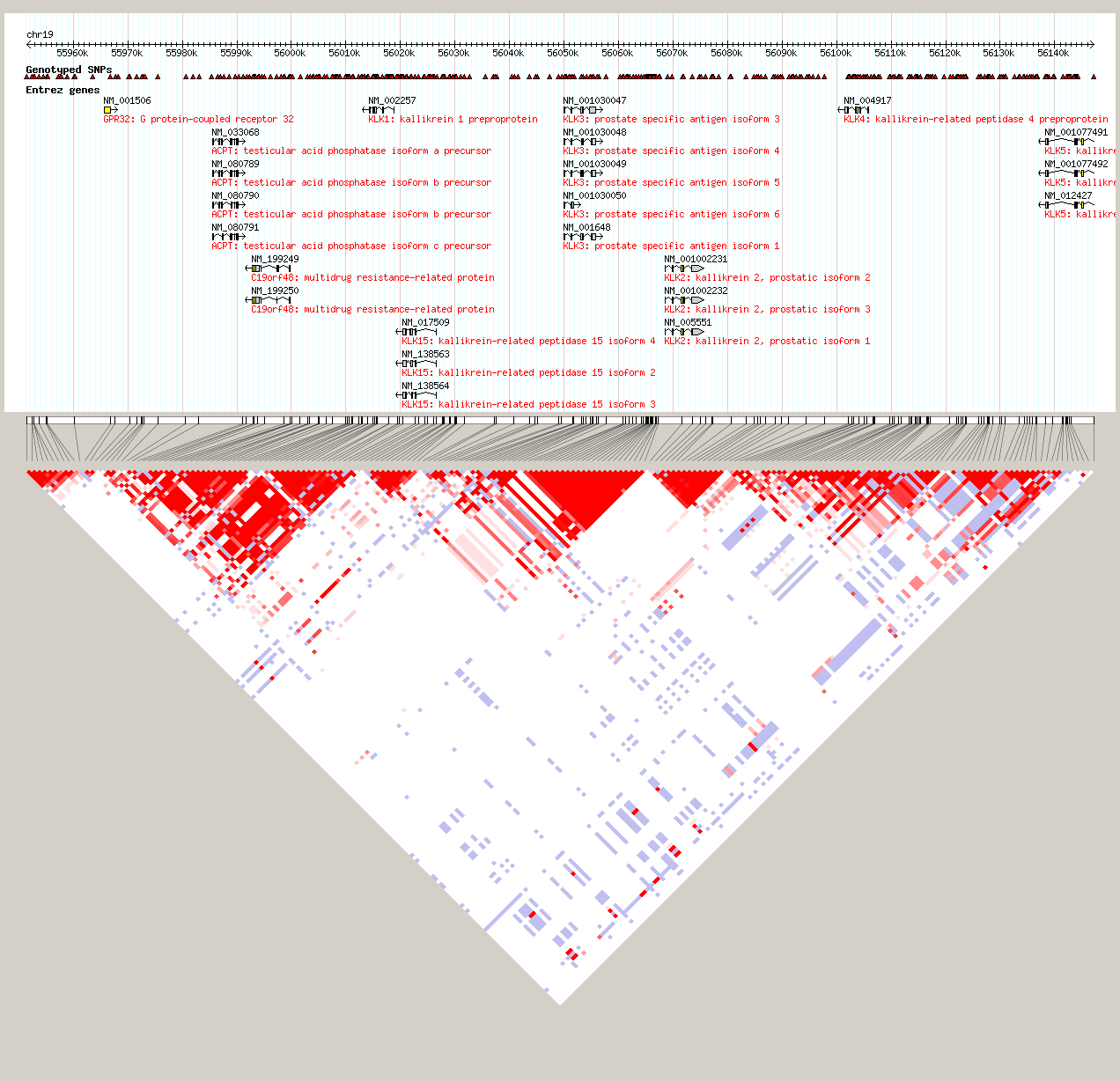
**

Signal in African Americans

Index Signals

**19q13, YRI HapMap Phase 2**

**
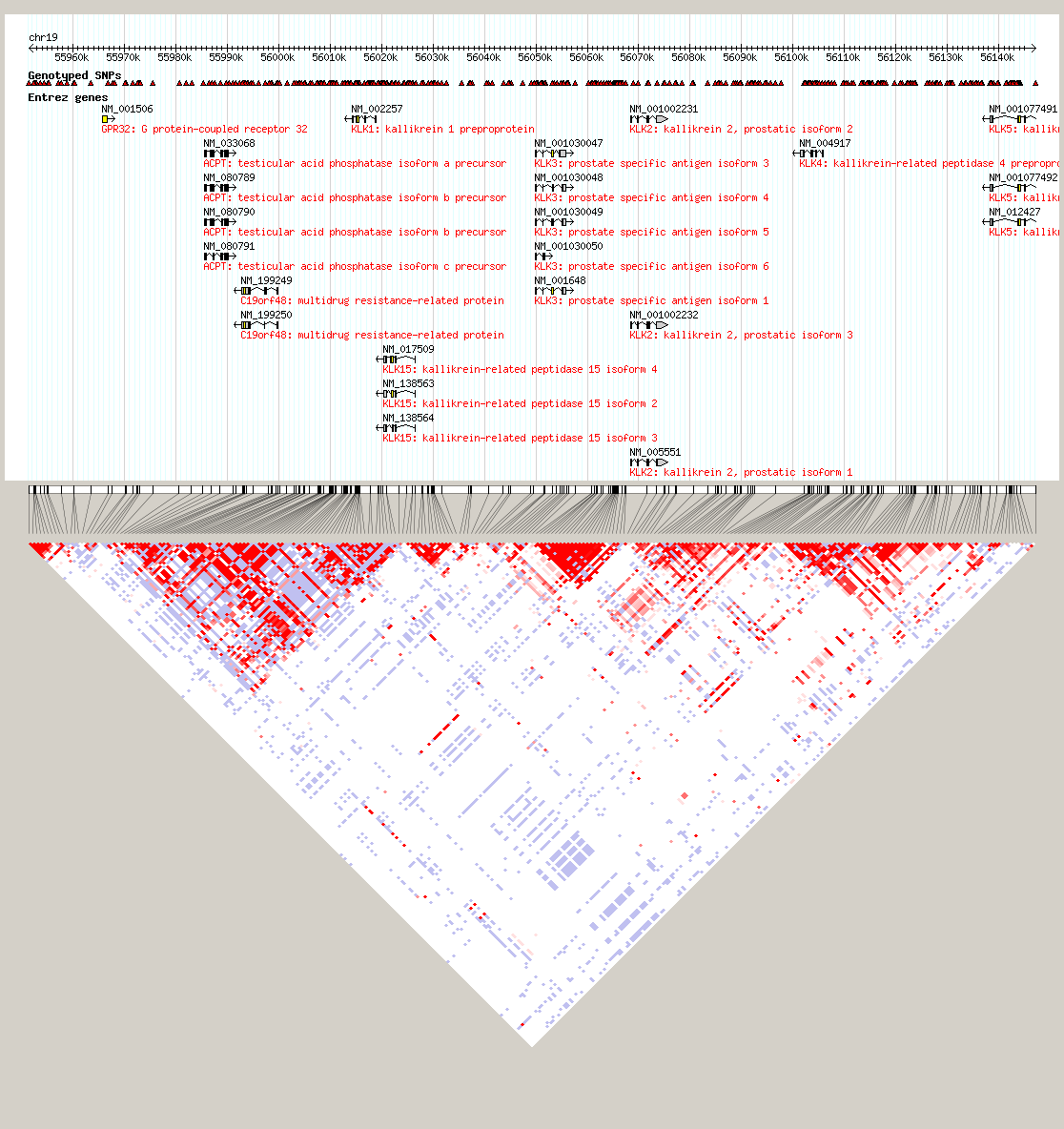
**

Signal in African Americans

Index Signals

**Xp11, CEU HapMap Phase 2**

**
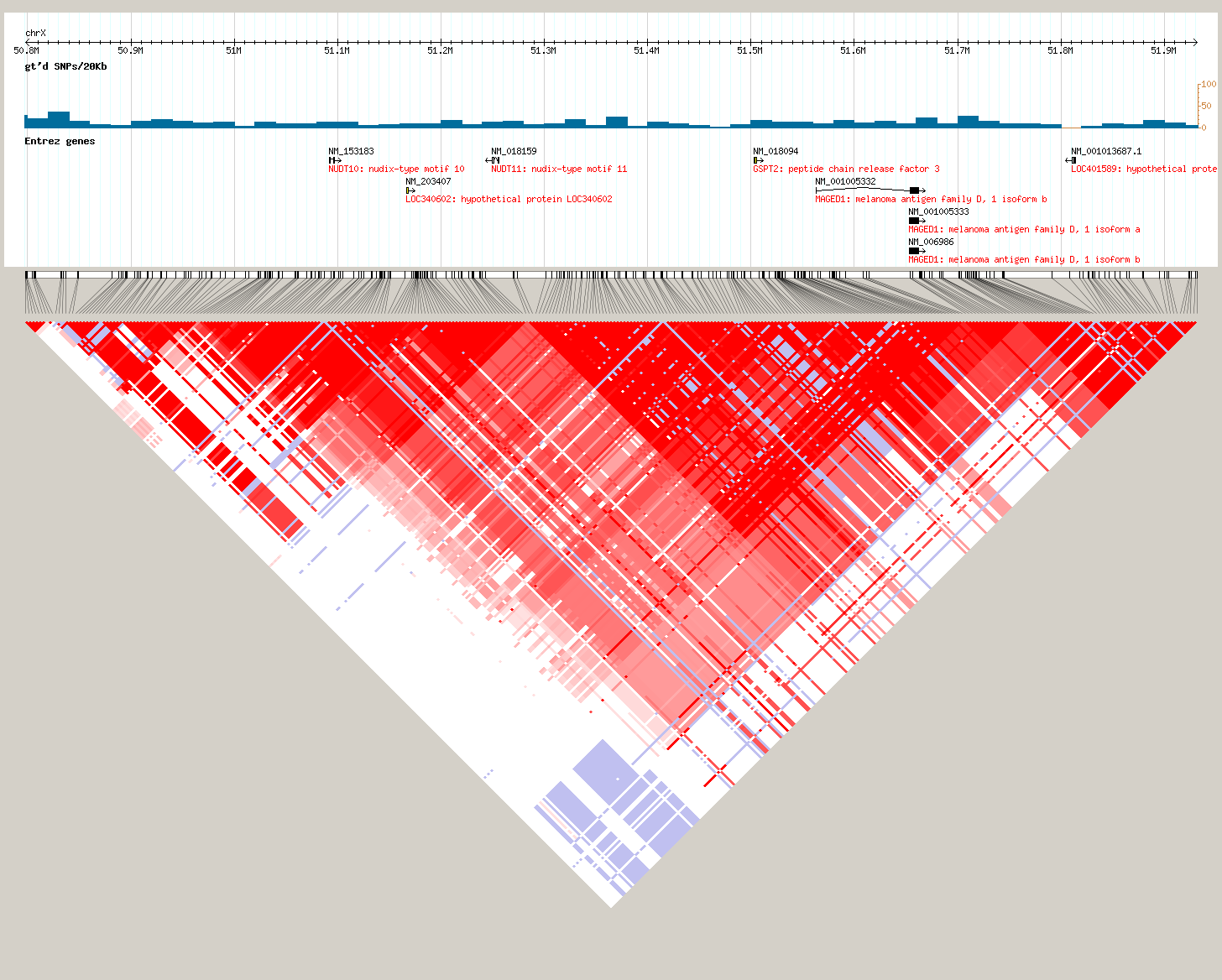
**

Signal in African Americans

Index Signals

**Xp11, YRI HapMap Phase 2**

**
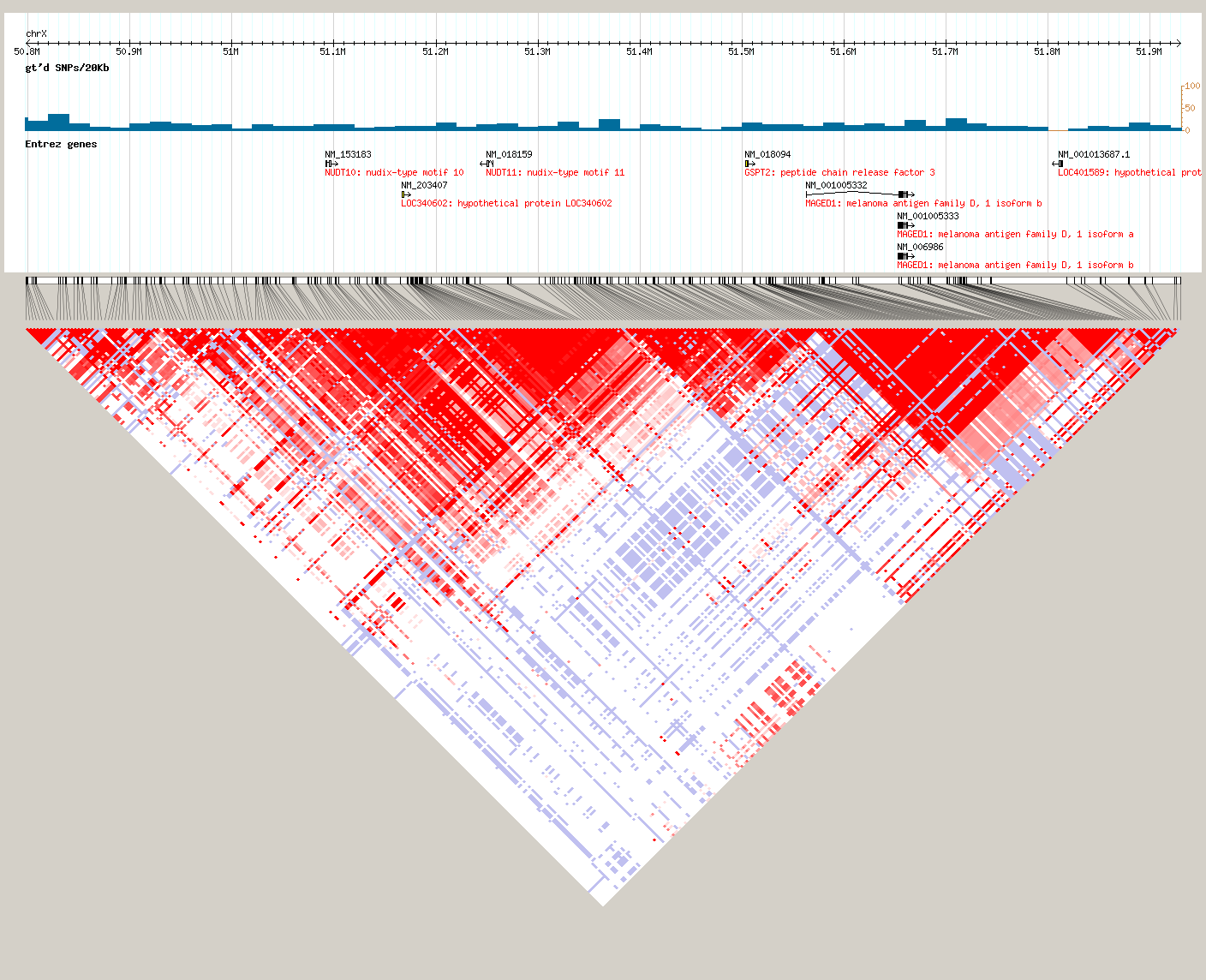
**

Index Signals

Signal in African Americans
