# Supplementary figures and images for: Characterizing Genetic Risk at Known Prostate Cancer Susceptibility Loci in African Americans
Source: PLoS Genet. 2011 May 26;7(5):e1001387. doi: 10.1371/journal.pgen.1001387 (PMC3102736; doi:10.1371/journal.pgen.1001387)

**
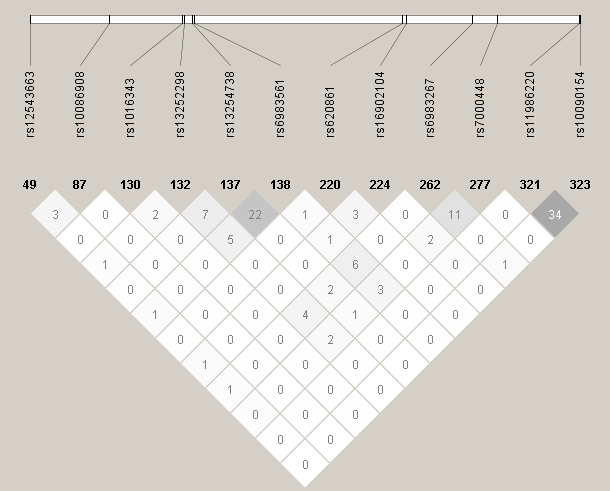
**

Supplement: Figure S2 — Pairwise correlation (r2) of known risk variants at 8q24 in African Americans estimated in 941 African Americans in the MEC. (0.04 MB DOCX) [file pgen.1001387.s002.docx]
