# Supplementary material for: Characterizing Genetic Risk at Known Prostate Cancer Susceptibility Loci in African Americans
Source: PLoS Genet. 2011 May 26;7(5):e1001387. doi: 10.1371/journal.pgen.1001387 (PMC3102736; doi:10.1371/journal.pgen.1001387)
Supplement: Table S1 — Descriptive characteristics of the 11 studies included in stage 1 of the GWAS of prostate cancer in African Americans. (0.02 MB DOCX) [file pgen.1001387.s003.docx]

**Table S1. Descriptive characteristics of the 11 studies included in stage 1 of the GWAS of prostate cancer in African Americans.**

| **Study** | **MEC** | **SCCS** | **PLCO** | **CPS-II** | **MDA** | **IPCG** | **LAAPC** | **APCS** | **DCPC** | **KCPCS** | **GECAP** | **TOTAL** |
| --- | --- | --- | --- | --- | --- | --- | --- | --- | --- | --- | --- | --- |
| **N**  Cases  Controls | 1060  1055 | 201  412 | 231  240 | 64  112 | 528  437 | 354  157 | 288  287 | 71  85 | 263  341 | 141  75 | 224  89 | 3425  3290 |
| **Age (median, yrs.)**  Cases  Controls | 70  70 | 61  59 | 68  63 | 70  71 | 60  58 | 57  52 | 63  64 | 67  66 | 64  58 | 59  53 | 62  62 | 65  64 |
| **Stage (n,%)**  Localized  Non-Localized | 902(85)  103(10) | 160(80)  27(13) | 202(87)  23(10) | 54(84)  6(9) | 419(79)  98(19) | 220(62)  116(33) | 158(55)  101(35) | 37(52)  26(37) | 20(8)  31(12) | 111(79)  30(21) | 185(83)  39(17) | 2468(72)  600(18) |
| **Grade (n,%)**  Gleason<5  Gleason5-7  Gleason 8+ | 59(6)  680(64)  268(25) | 4(2)  103(51)  81(40) | 6(3)  183(79)  34(15) | 2(3)  44(69)  5(8) | 1(<1)  366(69)  145(27) | 0(0)  320(90)  31(9) | 0(0)  215(75)  65(23) | 0(0)  57(80)  14(20) | 12(5)  100(38)  26(10) | 5(4)  120(85)  14(10) | 2(1)  190(85)  23(10) | 91(3)  2378(69)  706(21) |
| **Severity (n,%)^a^**  Non-Advanced  Advanced | 663(63)  322(30) | 95(47)  90(45) | 175(76)  48(21) | 43(67)  10(16) | 328(62)  183(35) | 212(60)  124(35) | 134(47)  136(47) | 32(45)  35(49) | 20(8)  49(17) | 101(72)  38(27) | 165(74)  52(23) | 1968(57)  1087(32) |

^a^Non-Advanced = localized stage and Gleason<8; Advanced = non-localized stage and/or Gleason 8-10.
