# Supplementary material for: Characterizing Genetic Risk at Known Prostate Cancer Susceptibility Loci in African Americans
Source: PLoS Genet. 2011 May 26;7(5):e1001387. doi: 10.1371/journal.pgen.1001387 (PMC3102736; doi:10.1371/journal.pgen.1001387)
Supplement: Table S2 — Power to detect associations with the known risk variants in African American. (0.05 MB DOCX) [file pgen.1001387.s004.docx]

**Table S2. Power to detect associations with the known risk variants in African American.**

| **Chr, Index SNP** | **Reported OR (per allele)** | **RAF in African Americans** | **Power (α=0.05)** | **Reference** |
| --- | --- | --- | --- | --- |
| 2p24,rs13385191 | 1.15^a^ | 0.06 | 51% | [1] |
| 2p21,rs1465618 | 1.08 | 0.12 | 31% | [2] |
| 2p15, rs721048 | 1.15 | 0.04 | 35% | [3] |
| 2p15,rs2710647 | 1.15 | 0.46 | >80%^d^ | [2] |
| 2q21,rs12621278 | 1.30 | 0.98 | 52% | [2] |
| 3p12,rs2660753 | 1.08 | 0.49 | 61% | [4] |
| 3q21,rs10934853 | 1.12 | 0.70 | 84% | [5] |
| 4q22,rs12500426 | 1.08 | 0.40 | 59% | [2] |
| 4q22,rs17021918 | 1.10 | 0.78 | 62% | [2] |
| 4q24,rs7679673 | 1.09 | 0.39 | 69% | [2] |
| 5p15,rs401681 | 1.07 | 0.41 | 49% | [6] |
| 5p15,rs12653946 | 1.26^a^ | 0.41 | >99% | [1] |
| 6p21,rs1983891 | 1.15^a^ | 0.48 | 98% | [1] |
| 6q22,rs339331 | 1.22^a^ | 0.75 | >99% | [1] |
| 6q25,rs9364554 | 1.14 | 0.06 | 46% | [4] |
| 7p15,rs10486567 | 1.12 | 0.71 | 84% | [7] |
| 7q21,rs6465657 | 1.12 | 0.87 | 58% | [4] |
| 8p21,rs2928679 | 1.05 | 0.27 | 24% | [2] |
| 8p21,rs1512268 | 1.18 | 0.63 | >99% | [2] |
| 8q24,rs12543663 | 1.08 | 0.15 | 36% | [8] |
| 8q24,rs10086908 | 1.15 | 0.75 | 93% | [8] |
| 8q24,rs1016343 | 1.21 | 0.22 | >99% | [8] |
| 8q24,rs13252298 | 1.19 | 0.93 | 70% | [8] |
| 8q24,rs13254738 | 1.18^b^ | 0.60 | >99% | [9] |
| 8q24,rs6983561 | 1.47 | 0.44 | >99% | [8] |
| 8q24,rs620861 | 1.11 | 0.65 | 82% | [8] |
| 8q24,rs16902104 | 1.21 | 0.07 | 83% | [5] |
| 8q24,rs6983267 | 1.26 | 0.88 | 99% | [8] |
| 8q24,rs7000448 | 1.19^b^ | 0.62 | >99% | [9] |
| 8q24,rs11986220 | 1.64^b^ | 0.05 | >99% | [10] |
| 8q24,rs10090154 | 1.47 | 0.13 | >99% | [8] |
| 10q11,rs10993994 | 1.25 | 0.60 | >99% | [4] |
| 10q26, rs4962416 | 1.17 | 0.16 | 93% | [7] |
| 11p15, rs7127900 | 1.22 | 0.36 | >99% | [2] |
| 11q13,rs12418451 | 1.15 | 0.13 | 80% | [11] |
| 11q13,rs11228565 | 1.23 | 0.10 | 96% | [5] |
| 11q13, rs7931342 | 1.18 | 0.78 | 97% | [4] |
| 11q13,rs10896449 | 1.18 | 0.67 | >99% | [7] |
| 13q22,rs9600079 | 1.18^a^ | 0.53 | >99% | [1] |
| 17p12, rs4054823^c^ | 1.13 | 0.68 | 90% | [12] |
| 17q12,rs11649743 | 1.28 | 0.91 | 97% | [13] |
| 17q12, rs4430796 | 1.24 | 0.35 | >99% | [14] |
| 17q12,rs7501939 | 1.19 | 0.49 | >99% | [14] |
| 17q24, rs1859962 | 1.25 | 0.30 | >99% | [14] |
| 19q13, rs8102476 | 1.12 | 0.74 | 81% | [5] |
| 19q13, rs266849 | 1.05 | 0.88 | 15% | [15] |
| 19q13, rs2735839 | 1.12 | 0.69 | >99% | [4] |
| 22q13, rs5759167 | 1.16 | 0.75 | 95% | [2] |
| Xp11, rs5945572 | 1.23 | 0.28 | >99% | [3] |

^a^Identified in Japanese. ^b^Estimated in a multiethnic sample. ^c^SNP for aggressive disease. ^d^Per allele OR not provided. Estimated based on OR for heterozygotes and homozygotes.

**References**

1. Takata R, Akamatsu S, Kubo M, Takahashi A, Hosono N, et al. Genome-wide association study identifies five new susceptibility loci for prostate cancer in the Japanese population. Nat Genet 42: 751-754.

2. Eeles RA, Kote-Jarai Z, Al Olama AA, Giles GG, Guy M, et al. (2009) Identification of seven new prostate cancer susceptibility loci through a genome-wide association study. Nat Genet 41: 1116-1121.

3. Gudmundsson J, Sulem P, Rafnar T, Bergthorsson JT, Manolescu A, et al. (2008) Common sequence variants on 2p15 and Xp11.22 confer susceptibility to prostate cancer. Nat Genet 40: 281-283.

4. Kote-Jarai Z, Easton DF, Stanford JL, Ostrander EA, Schleutker J, et al. (2008) Multiple novel prostate cancer predisposition loci confirmed by an international study: the PRACTICAL Consortium. Cancer Epidemiol Biomarkers Prev 17: 2052-2061.

5. Gudmundsson J, Sulem P, Gudbjartsson DF, Blondal T, Gylfason A, et al. (2009) Genome-wide association and replication studies identify four variants associated with prostate cancer susceptibility. Nat Genet 41: 1122-1126.

6. Rafnar T, Sulem P, Stacey SN, Geller F, Gudmundsson J, et al. (2009) Sequence variants at the TERT-CLPTM1L locus associate with many cancer types. Nat Genet 41: 221-227.

7. Thomas G, Jacobs KB, Yeager M, Kraft P, Wacholder S, et al. (2008) Multiple loci identified in a genome-wide association study of prostate cancer. Nat Genet 40: 310-315.

8. Al Olama AA, Kote-Jarai Z, Giles GG, Guy M, Morrison J, et al. (2009) Multiple loci on 8q24 associated with prostate cancer susceptibility. Nat Genet 41: 1058-1060.

9. Haiman CA, Patterson N, Freedman ML, Myers SR, Pike MC, et al. (2007) Multiple regions within 8q24 independently affect risk for prostate cancer. Nat Genet 39: 638-644.

10. Jia L, Landan G, Pomerantz M, Jaschek R, Herman P, et al. (2009) Functional enhancers at the gene-poor 8q24 cancer-linked locus. PLoS Genet 5: e1000597.

11. Zheng SL, Stevens VL, Wiklund F, Isaacs SD, Sun J, et al. (2009) Two independent prostate cancer risk-associated Loci at 11q13. Cancer Epidemiol Biomarkers Prev 18: 1815-1820.

12. Xu J, Zheng SL, Isaacs SD, Wiley KE, Wiklund F, et al. Inherited genetic variant predisposes to aggressive but not indolent prostate cancer. Proc Natl Acad Sci U S A 107: 2136-2140.

13. Sun J, Zheng SL, Wiklund F, Isaacs SD, Purcell LD, et al. (2008) Evidence for two independent prostate cancer risk-associated loci in the HNF1B gene at 17q12. Nat Genet 40: 1153-1155.

14. Gudmundsson J, Sulem P, Steinthorsdottir V, Bergthorsson JT, Thorleifsson G, et al. (2007) Two variants on chromosome 17 confer prostate cancer risk, and the one in TCF2 protects against type 2 diabetes. Nat Genet 39: 977-983.

15. Eeles RA, Kote-Jarai Z, Giles GG, Olama AA, Guy M, et al. (2008) Multiple newly identified loci associated with prostate cancer susceptibility. Nat Genet 40: 316-321.
