# Supplementary material for: Characterizing Genetic Risk at Known Prostate Cancer Susceptibility Loci in African Americans
Source: PLoS Genet. 2011 May 26;7(5):e1001387. doi: 10.1371/journal.pgen.1001387 (PMC3102736; doi:10.1371/journal.pgen.1001387)
Supplement: Table S3 — The association of local ancestry surrounding the index signal(s) at each risk locus and prostate cancer risk. (0.02 MB DOCX) [file pgen.1001387.s005.docx]

**Table S3. The association of local ancestry surrounding the index signal(s) at each risk locus and prostate cancer risk.**

| Chromosome Region | OR per European chromosome | P-value |
| --- | --- | --- |
| 2p24 | 0.94 | 0.18 |
| 2p21, | 0.96 | 0.34 |
| 2p15, | 0.93 | 0.13 |
| 2q21 | 0.98 | 0.74 |
| 3p12 | 1.06 | 0.10 |
| 3q21 | 0.94 | 0.17 |
| 4q22 | 1.04 | 0.40 |
| 4q24 | 1.03 | 0.57 |
| 5p15 | 1.02 | 0.75 |
| 5p15 | 1.02 | 0.68 |
| 6p21 | 0.95 | 0.28 |
| 6q22 | 1.09 | 0.057 |
| 6q25 | 0.94 | 0.22 |
| 7p15 | 1.16 | 1.6x10^-3^ |
| 7q21 | 0.99 | 0.87 |
| 8p21 | 0.91 | 0.051 |
| 8q24 | 0.81 | 4.7x10^-5^ |
| 10q11 | 0.98 | 0.68 |
| 10q26 | 1.14 | 6.2x10^-3^ |
| 11p15 | 1.09 | 0.079 |
| 11q13 | 0.96 | 0.43 |
| 13q22 | 0.98 | 0.73 |
| 17p12 | 1.04 | 0.42 |
| 17q12 | 1.00 | 0.97 |
| 17q24 | 0.97 | 0.50 |
| 19q13 | 0.99 | 0.84 |
| 19q13 | 1.09 | 0.084 |
| 22q13 | 0.88 | 0.01 |
| Xp11 | NA^a^ | NA |

^a^ Not available for the X chromosome.
