# Supplementary material for: Characterizing Genetic Risk at Known Prostate Cancer Susceptibility Loci in African Americans
Source: PLoS Genet. 2011 May 26;7(5):e1001387. doi: 10.1371/journal.pgen.1001387 (PMC3102736; doi:10.1371/journal.pgen.1001387)
Supplement: Table S4 — Associations with established risk variants for prostate cancer (3,425 cases, 3,290 controls) adjusted for global ancestry. (0.02 MB DOCX) [file pgen.1001387.s006.docx]

**Table S4. Associations with established risk variants for prostate cancer (3,425 cases, 3,290 controls) adjusted for global ancestry.**

| **Index SNP from GWAS** | |
| --- | --- |
| Chr., Marker Position, Alleles^a^ | RAF (EA/AA)^b^, OR (95% CI)^c^  P-value^d^ |
| 2p24,rs13385191 20,751,746,G/A | 0.61^f^/0.06, 0.95(0.82-1.10) 0.49 |
| 2p21,rs1465618  43,407,453,T/C | 0.23/0.12, 1.05(0.94-1.17) 0.37 |
| 2p15, rs721048  62,985,235,A/G | 0.19/0.04, 1.14(0.95-1.36) 0.15 |
| 2p15,rs2710647  63,067,474,C/T | 0.55/0.46, 1.15(1.08-1.24) 6.5x10^-5^ |
| 2q21,rs12621278  173,019,799,A/G | 0.94/0.98, 1.46(1.07-2.01) 0.018 |
| 3p12,rs2660753  87,193,364,T/C | 0.11/0.49, 0.97(0.90-1.04) 0.36 |
| 3q21,rs10934853  129,521,063,A/C | 0.28/0.70, 1.07(0.98-1.15) 0.11 |
| 4q22,rs12500426  95,733,632,A/C | 0.46/0.40, 1.00(0.94-1.08) 0.90 |
| 4q22,rs17021918  95,781,900,C/T | 0.66/0.78, 1.07(0.99-1.17) 0.10 |
| 4q24,rs7679673^e^  106,280,983,C/A | 0.55/0.39, 1.08(1.01-1.16)  0.031 |
| 5p15,rs401681  1,375,087,C/T | 0.55/0.41, 0.94(0.88-1.01) 0.083 |
| 5p15,rs12653946  1,948,829,T/C | 0.43^f^/0.41, 1.05(0.98-1.13) 0.15 |
| 6p21,rs1983891  41,644,405,T/C | 0.38^f^/0.48, 1.09(1.01-1.17) 0.018 |
| 6q22,rs339331  117,316,745,T/C | 0.63^f^/0.75, 1.21(1.12-1.32) 4.8x10^-6^ |
| 6q25,rs9364554  160,753,654,T/C | 0.29/0.06, 1.20(1.04-1.38) 0.011 |
| 7p15,rs10486567  27,943,088,G/A | 0.77/0.71, 1.15(1.07-1.25) 2.7x10^-4^ |
| 7q21,rs6465657  97,654,263,C/T | 0.46/0.87, 1.06(0.94-1.19) 0.37 |
| 8p21,rs2928679  23,494,920,A/G | 0.42/0.27, 1.01(0.93-1.09) 0.80 |
| 8p21,rs1512268  23,582,408,T/C | 0.45/0.63, 1.13(1.05-1.21) 1.3x10^-3^ |
| 10q11,rs10993994  51,219,502,T/C | 0.40/0.60, 1.09(1.01-1.17) 0.022 |
| 10q26, rs4962416  126,686,862,C/T | 0.27/0.16, 1.07(0.97-1.17) 0.16 |
| 11p15, rs7127900  2,190,150,A/G | 0.20/0.36, 1.07(1.00-1.15) 0.062 |
| 11q13,rs12418451^e^  68,691,995,A/G | 0.28/0.13, 1.12(1.00-1.25)  0.051 |
| 11q13,rs11228565  68,735,156,A/G | 0.20/0.10, 1.07(0.96-1.20) 0.23 |
| 11q13, rs7931342  68,751,073,G/T | 0.51/0.78, 1.13(1.04-1.23) 5.2x10^-3^ |
| 11q13,rs10896449 68,751,243,G/A | 0.52/0.67, 1.15(1.07-1.24) 2.3x10^-4^ |
| 13q22,rs9600079  72,626,140,T/G | 0.35^f^/0.52, 0.98(0.91-1.05) 0.55 |
| 17p12, rs4054823 13,565,749,T/C | 0.56/0.68, 0.98(0.91-1.06) 0.67 |
| 17q12,rs11649743 33,149,092,G/A | 0.80/0.91, 1.14(1.00-1.29) 0.051 |
| 17q12, rs4430796 33,172,153,A/G | 0.53/0.35, 1.03(0.95-1.10) 0.50 |
| 17q12,rs7501939  33,175,269,C/T | 0.58/0.49, 1.03(0.96-1.10) 0.43 |
| 17q24, rs1859962  66,620,348,G/T | 0.46/0.30, 0.99(0.92-1.06) 0.77 |
| 19q13, rs8102476 43,427,453,C/T | 0.54/0.74, 1.12(1.03-1.21) 8.2x10^-3^ |
| 19q13, rs266849 56,040,902,A/G | 0.80/0.88, 1.00(0.90-1.12) 0.99 |
| 19q13, rs2735839 56,056,435,G/A | 0.85/0.69, 0.95(0.88-1.03) 0.20 |
| 22q13, rs5759167 41,830,156,G/T | 0.53/0.75, 1.13(1.04-1.23) 4.0x10^-3^ |
| Xp11, rs5945572 51,246,423,A/G | 0.35/0.14, 1.21(1.09-1.35) 5.2x10^-4^ |

^a^Risk allele/reference allele. ^b^RAF, risk allele frequency in populations of European ancestry (EA) from previous reports or HapMap CEU population, and in African Americans (AA) in this study. ^c^Adjusted for age, study and the 1^st^ 10 eigenvalues. ^d^Test of trend (1-d.f.). ^e^Imputed. ^f^Index signal reported in GWAS in Japanese. RAF in Japanese.
