# Supplementary material for: Characterizing Genetic Risk at Known Prostate Cancer Susceptibility Loci in African Americans
Source: PLoS Genet. 2011 May 26;7(5):e1001387. doi: 10.1371/journal.pgen.1001387 (PMC3102736; doi:10.1371/journal.pgen.1001387)
Supplement: Table S5 — Information about the 27 regions fine-mapped (not including 8q24). (0.03 MB DOCX) [file pgen.1001387.s007.docx]

**Table S5. Information about the 27 regions fine-mapped (not including 8q24).**

| **Chr.** | **Index SNP** | **Position (MB)** | **Start** | **Stop** | **Size^a^**  **(Kb)** | **No. of common SNPs in Phase 2 HapMap (YRI)**  **(MAF≥0.05, HWE≥0.01)** | **No. of tags in YRI (r^2^≥0.8)** | **No. of typed and imputed SNPs tested (MAF≥0.01)** |
| --- | --- | --- | --- | --- | --- | --- | --- | --- |
| 2p24 | rs13385191^b^ | 20751746 | 20501746 | 21001746 | 500 | **629** | **364** | 749 |
| 2p21 | rs1465618 | 43407453 | 43157453 | 43857453 | 700 | **795** | **359** | 934 |
| 2p15 | rs721048 | 62985235 | 62535235 | 63435235 | 900 | **364** | **158** | 603 |
| 2p15 | rs2710647 | 63067474 | 62535235 | 63435235 | 900 | **364** | **158** | 603 |
| 2q21 | rs12621278 | 173019799 | 172769799 | 173269799 | 500 | **563** | **284** | 713 |
| 3p12 | rs2660753 | 87193364 | 86943364 | 87543364 | 600 | **535** | **120** | 628 |
| 3q21 | rs10934853 | 129521063 | 129171063 | 129771063 | 600 | **449** | **151** | 544 |
| 4q22 | rs12500426 | 95733632 | 95483632 | 95983632 | 500 | **380** | **182** | 499 |
| 4q22 | rs17021918 | 95781900 | 95483632 | 95983632 | 500 | **380** | **182** | 499 |
| 4q24 | rs7679673 | 106280983 | 106030983 | 106630983 | 600 | **333** | **171** | 536 |
| 5p15 | rs401681 | 1375087 | 1125087 | 1625087 | 500 | **308** | **217** | 455 |
| 5p15 | rs12653946^b^ | 1948829 | 1698829 | 2198829 | 500 | **663** | **415** | 803 |
| 6p21 | rs1983891^b^ | 41644405 | 41394405 | 41894405 | 500 | **403** | **261** | 495 |
| 6q22 | rs339331^b^ | 117316745 | 117066745 | 117566745 | 500 | **469** | **108** | 552 |
| 6q25 | rs9364554 | 160753654 | 160503654 | 161003654 | 500 | **468** | **190** | 603 |
| 7p15 | rs10486567 | 27943088 | 27443088 | 28193088 | 750 | **610** | **315** | 887 |
| 7q21 | rs6465657 | 97654263 | 97404263 | 98004263 | 600 | **344** | **170** | 440 |
| 8p21 | rs2928679 | 23494920 | 23244920 | 23832408 | 587 | **646** | **298** | 786 |
| 8p21 | rs1512268 | 23582408 | 23244920 | 23832408 | 587 | **646** | **298** | 786 |
| 10q11 | rs10993994 | 51219502 | 50769502 | 51469502 | 700 | **99** | **50** | 130 |
| 10q26 | rs4962416 | 126686862 | 126436862 | 126936862 | 500 | **397** | **244** | 543 |
| 11p15 | rs7127900 | 2190150 | 1940150 | 2440150 | 500 | **370** | **231** | 551 |
| 11q13 | rs12418451 | 68691995 | 68441995 | 69001243 | 559 | **535** | **308** | 678 |
| 11q13 | rs11228565 | 68735156 | 68441995 | 69001243 | 559 | **535** | **308** | 678 |
| 11q13 | rs7931342 | 68751073 | 68441995 | 69001243 | 559 | **535** | **308** | 678 |
| 11q13 | rs10896449 | 68751243 | 68441995 | 69001243 | 559 | **535** | **308** | 678 |
| 13q22 | rs9600079^b^ | 72626140 | 72376140 | 72876140 | 500 | **503** | **243** | 647 |
| 17p12 | rs4054823 | 13565749 | 13315749 | 13815749 | 500 | **666** | **383** | 819 |
| 17q12 | rs11649743 | 33149092 | 32899092 | 33425269 | 526 | **262** | **185** | 377 |
| 17q12 | rs4430796 | 33172153 | 32899092 | 33425269 | 526 | **262** | **185** | 377 |
| 17q12 | rs7501939 | 33175269 | 32899092 | 33425269 | 526 | **262** | **185** | 377 |
| 17q24 | rs1859962 | 66620348 | 66370348 | 66870348 | 500 | **569** | **301** | 711 |
| 19q13 | rs8102476 | 43427453 | 43177453 | 43677453 | 500 | **239** | **115** | 269 |
| 19q13 | rs266849 | 56040902 | 55790902 | 56306435 | 516 | **511** | **344** | 652 |
| 19q13 | rs2735839 | 56056435 | 55790902 | 56306435 | 516 | **511** | **344** | 652 |
| 22q13 | rs5759167 | 41830156 | 41580156 | 42080156 | 500 | **559** | **342** | 742 |
| Xp11 | rs5945572 | 51246423 | 50796423 | 52096423 | 1300 | **338** | **83** | 560 |

^a^Target region was 250kb on either side of the index SNP. The region was expanded in cases where the LD block containing the index SNP was >250kb. ^b^Variants identified in Japanese. LD estimates based on HapMap JPT/CHB populations. ^c^Index SNP not in Phase 2 HapMap.
