# Supplementary material for: Characterizing Genetic Risk at Known Prostate Cancer Susceptibility Loci in African Americans
Source: PLoS Genet. 2011 May 26;7(5):e1001387. doi: 10.1371/journal.pgen.1001387 (PMC3102736; doi:10.1371/journal.pgen.1001387)
Supplement: Table S6 — Results of the stepwise procedure in each risk region (not including 8q24). (0.02 MB DOCX) [file pgen.1001387.s008.docx]

**Table S6. Results of the stepwise procedure in each risk region (not including 8q24).**

| **Region** | **SNPs Selected^a^** | **Correlated with Index (r^2^>0.2)** |
| --- | --- | --- |
| 2p24 | rs340623 | Yes |
| 2p21 | - |  |
| 2p15 | rs6545977 | Yes |
| 2q21 | rs12620581 | Yes |
| 3p12 | - |  |
| 3q21 | rs7641133 | Yes |
| 4q22 | - |  |
| 4q24 | - |  |
| 5p15 | - |  |
| 6p21 | - |  |
| 6q22 | rs12202378 | Yes |
| 6q25 | rs2076828 | Yes |
| 7p15 | rs7808935 | Yes |
| 7q21 | - |  |
| 8p21 | rs11782388 | Yes |
| 10q11 | rs4630243 | Yes |
| 10q26 | - |  |
| 11p15 | - |  |
| 11q13 | rs11228580 | Yes |
| 13q22 | - |  |
| 17p12 | - |  |
| 17q12 | - |  |
| 17q24 | - |  |
| 19q13 | - |  |
| 19q13 | rs3760722 | Yes |
| 22q13 | - |  |
| Xp11 | rs4907796 | Yes |

^a^In each region, markers correlated with the index signal were kept in the model if p<0.004, all other markers were kept if p<5.6x10^-6^.
