# Supplementary material for: Characterizing Genetic Risk at Known Prostate Cancer Susceptibility Loci in African Americans
Source: PLoS Genet. 2011 May 26;7(5):e1001387. doi: 10.1371/journal.pgen.1001387 (PMC3102736; doi:10.1371/journal.pgen.1001387)
Supplement: Table S7 — Associations by genotype class for SNPs in known prostate cancer risk regions that were found to be nominally associated with risk in African Americans. (0.02 MB DOCX) [file pgen.1001387.s009.docx]

| Chr., Marker Position, Alleles | RAF^a^ in African Americans | Per allele  OR (95% CI)^b^ | Heterozygotes  OR (95% CI)^b^ | Homozygotes  OR (95% CI)^b^ |
| --- | --- | --- | --- | --- |
| 2p24, rs340623^c^  20,795,759,C/T | 0.17 | 1.15(1.05-1.25) | 1.17(1.04-1.31) | 1.20(0.91-1.58) |
| 2p15,rs6545977 63,154,668,G/A | 0.48 | 1.18(1.10-1.27) | 1.14(1.01-1.28) | 1.40(1.22-1.61) |
| 2q21,rs12620581^c^ 173,037,960,A/G | 0.75 | 1.13(1.03-1.23) | 1.01(0.81-1.26) | 1.18(0.95-1.46) |
| 3q21,rs7641133  129,319,009, T/C | 0.29 | 1.16(1.08-1.25) | 1.17(1.06-1.30) | 1.33(1.11-1.58) |
| 4q24,rs7679673^c^  106,280,983,C/A | 0.39 | 1.08(1.01-1.16) | 1.01(0.91-1.13) | 1.19(1.03-1.39) |
| 6p21, rs1983891 41,644,405, T/C | 0.48 | 1.09(1.01-1.17) | 1.05(0.93-1.19) | 1.18(1.02-1.36) |
| 6q22,rs12202378^c^ 117,348,714,T/C | 0.70 | 1.25(1.15-1.35) | 1.04(0.85-1.27) | 1.36(1.12-1.65) |
| 6q25,rs2076828 160,792,776,C/G | 0.56 | 1.14(1.06-1.22) | 1.18(1.03-1.35) | 1.31(1.13-1.51) |
| 7p15,rs7808935^c^ 27,943,888,T/C | 0.70 | 1.16(1.07-1.25) | 1.30(1.07-1.58) | 1.44(1.19-1.74) |
| 8p21,rs11782388^c^ 23,581,303,C/T | 0.70 | 1.18(1.09-1.28) | 1.26(1.04-1.53) | 1.44(1.18-1.75) |
| 10q11,rs4630243^c^ 51,210,873,T/C | 0.76 | 1.14(1.05-1.25) | 1.00(0.80-1.24) | 1.19(0.95-1.48) |
| 11p15,rs7127900 2,190,150,A/G | 0.36 | 1.09(1.01-1.17) | 1.13(1.01-1.25) | 1.15(00.98-1.35) |
| 11q13,rs12418451^c^  68,691,995,A/G | 0.13 | 1.13(1.01-1.27) | 1.13(1.00-1.27) | 1.08(0.73-1.60) |
| 11q13,rs11228580^c^ 68,758,918,C/T | 0.15 | 1.31(1.20-1.44) | 1.31(1.17-1.46) | 1.70(1.27-2.26) |
| 17q12,rs11649743  33,149,092,G/A | 0.91 | 1.15(1.01-1.31) | 0.89(0.45-1.77) | 1.04(0.53-2.06) |
| 19q13,rs8102476 43,427,453,C/T | 0.74 | 1.12(1.03-1.21) | 1.18(0.96-1.47) | 1.30(1.05-1.61) |
| 19q13,rs3760722 56,049,628,C/T | 0.72 | 1.14(1.05-1.24) | 1.06(0.86-1.30) | 1.24(1.01-1.52) |
| 22q13,rs5759167 41,830,156,G/T | 0.75 | 1.10(1.01-1.20) | 1.09(0.87-1.37) | 1.21(0.97-1.51) |
| Xp11,rs4907796 51,277,989,T/C | 0.13 | 1.25(1.12-1.39) | - | - |

**Table S7. Associations by genotype class for SNPs in known prostate cancer risk regions that were found to be nominally associated with risk in African Americans.**

^a^RAF, risk allele frequency. ^b^Adjusted for age, study, the 1^st^ 10 eigenvalues and local ancestry. ^c^For imputed SNPs, the probability of the number of risk alleles from MACH was converted to genotype groups (i.e. <0.5 = homozygous reference; 0.5-1.5 =heterozygous, and; >1.5=homozygous variant).
