# Supplementary material for: Characterizing Genetic Risk at Known Prostate Cancer Susceptibility Loci in African Americans
Source: PLoS Genet. 2011 May 26;7(5):e1001387. doi: 10.1371/journal.pgen.1001387 (PMC3102736; doi:10.1371/journal.pgen.1001387)
Supplement: Table S8 — Correlations (r2) between risk markers in African Africans and known risk variants at 8q24. (0.02 MB DOCX) [file pgen.1001387.s010.docx]

**Table S8. Correlations (r^2^) between risk markers in African Africans and known risk variants at 8q24.**

| **Position** | **SNP** | **Notes:** |
| --- | --- | --- |
| 127994810 | rs7839365 | 0.06 with rs12543663 in CEU, 0.10 with rs10086908 in CEU, 0.20 with rs12543663 in YRI, 0.07 with rs10086908 in YRI, 0.08 with rs6983561 in YRI |
| 128059437 | rs753228 | 0.11 with rs6983267 in YRI |
| 128162723 | rs4871008 | 0.26 with rs1016343 in CEU, 0.13 with rs1016343 in YRI |
| 128173119 | rs1456315 | 0.43 with rs1016343 in CEU, 0.23 with rs13252298 in CEU, 0.70 with rs13254738 in CEU, 0.41 with rs13254738 in YRI, 0.28 with rs6983561 in YRI |
| 128200973 | rs10098156 | 0.15 with rs13254738 in CEU, 0.12 with rs13254738 in YRI |
| 128219343 | rs6987409 | 0.06 with rs13254738 in YRI, 0.09 with rs6983561 in YRI |
| 128528307 | rs13282506 | 0.28 with rs6983267 in CEU, 0.20 with rs7000448 in CEU, 0.07 with rs6983267 in YRI, 0.15 with rs7000448 in YRI |
| 128589355 | rs7812429 | 1.0 with rs10090154 in CEU, 0.42 with rs10090154 in YRI |
| 128640941 | rs4313118 | No correlated SNPs |
