# Supplementary material for: Characterizing Genetic Risk at Known Prostate Cancer Susceptibility Loci in African Americans
Source: PLoS Genet. 2011 May 26;7(5):e1001387. doi: 10.1371/journal.pgen.1001387 (PMC3102736; doi:10.1371/journal.pgen.1001387)
Supplement: Table S9 — Independence of markers utilized in risk modeling. (0.02 MB DOCX) [file pgen.1001387.s011.docx]

**Table S9. Independence of markers utilized in risk modeling.**

| SNP | OR(95% CI)^a^ | OR(95%CI)^b^ |
| --- | --- | --- |
| rs340623 | 1.15(1.05-1.27) | 1.13(1.03-1.24) |
| rs6545977 | 1.18(1.10-1.27) | 1.19(1.10-1.27) |
| rs12620581 | 1.13(1.04-1.23) | 1.13(1.04-1.23) |
| rs7641133 | 1.16(1.08-1.25) | 1.16(1.08-1.25) |
| rs7679673 | 1.08(1.00-1.16) | 1.08(1.00-1.16) |
| rs1983891 | 1.09(1.01-1.17) | 1.10(1.02-1.18) |
| rs12202378 | 1.25(1.15-1.35) | 1.24(1.15-1.34) |
| rs2076828 | 1.14(1.06-1.22) | 1.13(1.05-1.22) |
| rs7808935 | 1.16(1.07-1.25) | 1.15(1.06-1.24) |
| rs11782388 | 1.18(1.09-1.28) | 1.19(1.09-1.28) |
| rs4630243 | 1.14(1.05-1.25) | 1.13(1.04-1.23) |
| rs7127900 | 1.09(1.01-1.17) | 1.08(1.00-1.16) |
| rs12418451 | 1.13(1.01-1.27) | 1.09(0.98-1.21) |
| rs11228580 | 1.31(1.20-1.44) | 1.31(1.19-1.44) |
| rs11649743 | 1.15(1.01-1.31) | 1.13(0.99-1.29) |
| rs8102476 | 1.12(1.03-1.21) | 1.12(1.03-1.22) |
| rs3760722 | 1.14(1.05-1.24) | 1.14(1.05-1.24) |
| rs5759167 | 1.10(1.01-1.20) | 1.13(1.04-1.23) |
| rs4907796 | 1.25(1.12-1.39) | 1.24(1.11-1.38) |

^a^Adjusted for age, study, the 1^st^ 10 eigenvalues and local ancestry. ^b^All SNPs in the same model; adjusted for age, study, the 1^st^ 10 eigenvalues and local ancestry.
